# Lightning assimilation in the WRF model (Version 4.1.1): technique updates and assessment of the applications from regional to hemispheric scales

**DOI:** 10.5194/gmd-15-8561-2022

**Published:** 2022-11-23

**Authors:** Daiwen Kang, Nicholas K. Heath, Robert C. Gilliam, Tanya L. Spero, Jonathan E. Pleim

**Affiliations:** 1 Center for Environmental Measurement and Modeling, Office of Research and Development, U.S. Environmental Protection Agency, Research Triangle Park, NC 27711, USA; 2 Air Quality and Atmospheric Composition, Atmospheric and Environmental Research, Lexington, MA 02421, USA

## Abstract

The lightning assimilation (LTA) technique in the Kain–Fritsch convective parameterization in the Weather Research and Forecasting (WRF) model has been updated and applied to continental and hemispheric simulations using lightning flash data obtained from the National Lightning Detection Network (NLDN) and the World Wide Lightning Location Network (WWLLN), respectively. The LTA technique uses lightning data to trigger the Kain–Fritsch convective parameterization via realistic temperature and moisture perturbations. The impact of different values for cumulus parameters associated with the Kain–Fritsch scheme on simulations with and without LTA were evaluated for both the continental and the hemispheric simulations. Comparisons to gauge-based rainfall products and near-surface meteorological observations indicated that the LTA improved the model’s performance for most variables. The simulated precipitation with LTA, using WWLLN lightning flashes in the hemispheric applications, was significantly improved over the simulations without LTA when compared to precipitation from satellite observations in the equatorial regions. The simulations without LTA showed significant sensitivity to the cumulus parameters (i.e., user-toggled switches) for monthly precipitation that was as large as 40 % during convective seasons for monthly mean daily precipitation. With LTA, the differences in simulated precipitation due to the different cumulus parameters were minimized. The horizontal grid spacing of the modeling domain strongly influenced the LTA technique and the predicted total precipitation, especially in the coarser scales used for the hemispheric simulation. The user-definable cumulus parameters and domain resolution manifested the complexity of convective process modeling both with and without LTA. These results revealed sensitivities to domain resolution, geographic heterogeneity, and the source and quality of the lightning dataset.

## 1 Introduction

Thunderstorms are natural phenomena that have intrigued human imagination for thousands of years. Although early efforts in atmospheric science and modeling were focused on understanding and forecasting thunderstorms, they remain difficult to accurately simulate in meteorological models. A variety of lightning parameterization schemes have been developed in regional and global atmospheric models (Price and Rind, 1992; Romps et al., 2014; Finney et al., 2014; Lopez, 2016), based on various physical, dynamical, and cloud properties, but these schemes marginally reproduce the spatial and temporal variability in lightning flashes with varying success over different regions of the globe. With the advancement of lightning detection technologies both at ground level and via satellite in the past decades, observed lightning flashes with coverage from regional to global scales are available and can be used for lightning assimilation (LTA). A robust LTA can improve convective simulations in meteorological models for retrospective atmospheric simulations (e.g., Heath et al., 2016; Marchand and Fuelberg, 2015) or help generate better initial fields for real-time weather forecasting (e.g., Lagouvardos et al., 2013; Giannaros et al., 2016; Fierro et al., 2012, 2015) by pinpointing where deep convection occurred and altering the meteorology in what is generally referred to as a hot start (Gan et al., 2021). In addition, lightning also profoundly impacts the chemical composition of the troposphere by generating and releasing nitrogen oxides (LNO_*x*_) that can significantly alter ground-level ozone (O_3_) concentrations in some regions (Kang et al., 2020). Because meteorological models drive air quality simulations, improving meteorological variables with LTA will cascade to chemistry fields simulated by air quality models (Allen et al., 2012; Kang et al., 2019a, b). It is especially critical when LNO_*x*_ emissions are included in air quality models, since LTA is designed to align LNO_*x*_ emissions with the time and location at which atmospheric convection occurred in the model, so the subsequent chemistry reactions and transport will more accurately reflect the emissions from lightning (Kang et al., 2019a, b).

Heath et al. (2016) implemented an LTA technique in the Kain–Fritsch (KF) convective scheme (Kain, 2004) in the Weather Research and Forecasting (WRF) model, which extended the works of Rogers et al. (2000), Mansell et al. (2007), Lagouvardos et al. (2013), and Giannaros et al. (2016). In general, the lightning assimilation approach is straightforward, activating deep convection where lightning is observed and only allowing shallow convection where it is not. Specifically, the LTA technique uses temperature and moisture perturbations to trigger KF deep convection where lightning is observed, resulting in a parameterized cloud with realistic characteristics based on the local environment and our understanding of lightning-producing convective clouds. It was tested using WRFv3.8 simulations for several months in 2011 using lightning observations from the National Lightning Detection Network (NLDN) over the contiguous United States (CONUS). It was found that the simulation of warm-season rainfall was substantially improved, and other near-surface meteorological variables were clearly improved in retrospective WRF applications. The LTA technique has been implemented in subsequent WRF releases (not publicly available yet) and applied in many meteorology and air quality studies over the CONUS (e.g., U.S. EPA, 2019; Appel et al., 2021). Although using LTA improved the predicted meteorological variables, some occasional unwanted departures from base model predictions without LTA occurred. Most commonly, LTA resulted in a low bias in summertime rainfall in some regions (U.S. EPA, 2019).

For this reason, it is of interest to investigate two parameters associated with the KF convective scheme with different optional values, which are specified in the WRF runtime name list input file and are often encountered by WRF users (https://www2.mmm.ucar.edu/wrf/users/docs/user_guide_v4/contents.html, last access: 7 November 2022). One parameter is called kfeta_trigger (also referred to as trigger, for simplicity, in this paper), which controls the conditions to determine how the KF convective scheme is triggered with three optional values, i.e, 1, the default value, 2, the moisture-advection-based trigger (only for ARW – the advanced research WRF dynamical solver), and 3, the relative humidity (RH)-dependent additional perturbation to option 1 (not tested). Another parameter is called cudt (namely cumulus time interval, delta t), and its value determines the minutes between cumulus physics calls (here it is the KF scheme). The default value of 0 indicates that the cumulus physics is called at every model step, and any non-zero value specifies the interval (min) that the cumulus physics is called (for example, cudt = 10 means that the cumulus physics is called every 10 min). Even though there are some discussions and recommendations regarding the choice of these parameter values through online forums or the WRF user mailing list (e.g., https://forum.mmm.ucar.edu/, last access: 7 November 2022; https://wrfems.info/, last access: 9 November 2022; https://www.epa.gov/sites/default/files/2017-02/documents/wrf_with_ltga_userguide.pdf, last access: 9 November 2022), there is no literature evaluating how these parameter values impact model performance when LTA is used.

The applications and evaluations of the LTA technique were limited to the CONUS, reflecting the areal coverage of NLDN (Murphy et al., 2021). As the spatial applications of atmospheric composition modeling are expanded from regional to hemispheric and global scales and new lightning datasets are available, there is a strong need to examine how this LTA technique performs at these larger scales when lightning flash data from a less accurate detection network are used. Thus, lightning flashes from the World Wide Lightning Location Network (WWLLN, operated by the University of Washington; http://www.wwlln.com, last access: 7 November 2022) is a suitable candidate because it has global coverage, albeit that its detection efficiency is lower than the > 95 % of NLDN for cloud-to-ground (CG) flashes (Abarca et al., 2010).

Our research has multiple objectives based on the aforementioned open research needs. We aim to (1) assess the impact of the parameter values associated with the KF convective scheme on WRF performance over the CONUS domain without LTA (base case) and with LTA using lightning flashes from NLDN, (2) examine the LTA in WRF using lightning flashes from WWLLN and compare to the simulations with NLDN lightning flashes, and (3) apply LTA to WRF simulations over the Northern Hemisphere and evaluate the performance in terms of precipitation and near-surface meteorological variables. In Sect. 2, we describe the updates made to the initial LTA technique (Heath et al., 2016). Section 3 provides the detailed data and methodologies of the model simulations and their evaluation. Section 4 presents our analysis on the impact of parameters with KF convective schemes with and without lightning assimilation over CONUS using lightning flashes from NLDN and WWLLN. In Sect. 5, we analyze the use of lightning flashes from WWLLN for LTA and evaluate WRF simulations with and without LTA over the Northern Hemisphere. And we conclude with key findings and recommendations in Sect. 6.

## 2 Updates on the LTA technique

The lightning assimilation used here is based on Heath et al. (2016), and a full description of the method can be found in that paper. Here, we provide only the essential details along with recent modifications to the scheme.

First, the lightning data (WWLLN or NLDN) are binned to the WRF domain in both time and space. The temporal binning is done every 30 min and includes lightning data from −10 to +20 min of the current time. The spatial regridding searches for a lightning strike within each grid box (using the staggered grid edge coordinates) within each time bin. This process creates a new lightning file with the same horizontal dimensions as the WRF domain filled with zeros (no lightning) or ones (lightning) at each 30 min time step. During the WRF simulation, if lightning is present, the scheme first goes through its standard updraft calculations, except that it uses the layer with the greatest moist static energy as its updraft source layer (USL). If the resulting cloud does not meet the criteria for deep convection, 0.1 g kg^−1^ of water vapor and 0.1 K are incrementally added to the USL until deep convection is forced. In the original Heath et al. (2016) scheme, only moisture was added to the USL. We have included temperature perturbations to further promote activating deep convection in these grid points with lightning.

In the unmodified KF scheme, a cloud must exceed a minimum depth (as a function of cloud base temperature) to satisfy the deep convection criteria. Specifically, a cloud base temperature greater than 20 °C must have a cloud greater than 4 km deep. For a cloud base temperature less than 0 °C, the cloud depth only needs to be 2 km. For cloud bases between 0 and 20 °C, the minimum cloud depth is defined as 2000 + 100*T*_LCL_, where *T*_LCL_ is the temperature at the lifted condensation level (LCL; Kain, 2004). Heath et al. (2016) modified this depth for lightning assimilation to be more consistent with lightning-producing storms. Specifically, within WRF, storms with a base temperature greater than or equal to 20 °C must have a cloud depth of at least 6 km, with a cloud top temperature less than −20 °C. Similarly, in the original model in Heath et al. (2016), storms with a cloud base temperature less than 20 °C must have a cloud depth of at least 4 km and a cloud top temperature less than −20 °C. These criteria were set to ensure that subgrid deep convective clouds were deep enough to have a mixed-phase layer to support lightning (e.g., Mansell et al., 2007; Bruning et al., 2014; Preston and Fuelberg, 2015). In this study, we slightly modified the scheme to require that the cloud top is at least one model level above the −20 °C level, ensuring that cloud-top temperatures are less than −20 °C (e.g., Stolzenburg and Marshall, 2009). The prior limit at −20 °C could inadvertently weaken simulated deep convective clouds, which may contribute to the dry bias in earlier applications of lightning assimilation approaches (U.S. EPA, 2019).

In Heath et al. (2016), if deep convection could not be achieved after incrementally adding up to 1 g kg^−1^ to the USL (which is now 1 g kg^−1^ and 1 K in our update), then no further action was taken, and deep convection was not activated by KF. However, to increase the realism of the scheme and increase the odds of deep convection the next time the scheme is called, we have updated the approach as follows. If a deep convective cloud cannot be activated, then the tallest cloud created is passed into the KF shallow convection scheme. In the KF scheme, shallow clouds are re-diagnosed each time the scheme is called. For example, suppose a shallow cloud is generated at *t* = 0, and KF is called at 5 min intervals. In that case, at the *t* = 5 min call, KF would determine if a shallow cloud is still present. Thus, the cloud can evolve so that, at *t* = 5 min, it could have slightly different characteristics than the one diagnosed at *t* = 0. This allows shallow clouds to grow, decay, or persist at short timescales.

Therefore, if the LTA method cannot trigger deep convection, the shallow cloud that is generated within WRF can precondition the atmosphere, thus increasing the likelihood of deep convection the next time the KF scheme with LTA is called. Therefore, these refinements to the LTA scheme in KF more closely replicate how convective initiation is observed in nature, where shallow cumulus and congestus clouds precondition the environment prior to deep convection initiation.

Finally, at grid points without observed lightning, deep convection is suppressed in WRF, and only the shallow portion of KF is allowed to run (this is referred to as the “ShallowOnly” method). Because convective clouds in nature can form and precipitate without generating lightning, this suppression technique serves as a realistic approach to reproduce nature given the constraints of the KF parameterization.

## 3 Data and methodology

### 3.1 Lightning flash data

**Figure 1.**
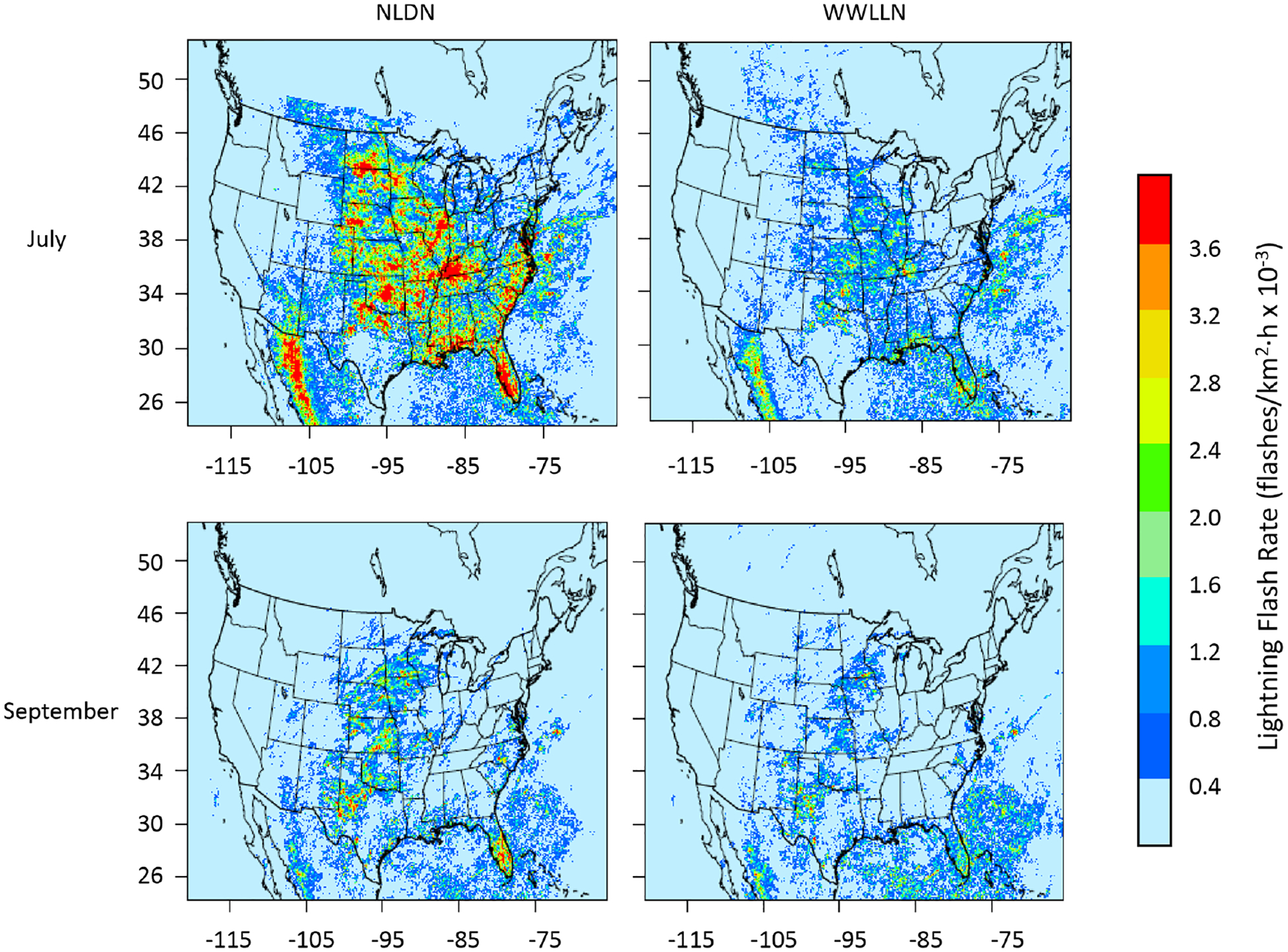
The mean hourly lightning flash rate from NLDN and WWLLN over the 12 km CONUS domain in July and September 2016.

Lightning flash data from two ground-based lightning detection networks were used for the assimilation using the LTA technique in this study. The NLDN provides cloud-to-ground lightning observations with a detection efficiency of > 95 % and a location accuracy of about 150 m (Murphy et al., 2021) over the contiguous U.S. (CONUS). The WWLLN provides global lightning data with lower detection efficiency and location accuracy (Abarca et al., 2010; Rudlosky and Shea, 2013; Burgesser, 2017) compared to NLDN and the Lightning Imaging Sensor (LIS) observations (Mach et al., 2007). Since WWLLN has global coverage, even with its relatively lower detection efficiency and location accuracy compared to NLDN, it could be a good option for applications beyond CONUS. Figure 1 shows how the average lightning flash rate (flashes km^−2^ h^−1^) from WWLLN compares to NLDN during July and September 2016 when hourly lightning flash counts are gridded into the CONUS 12 km grid cells.

As shown in Fig. 1, the lightning flash rates in NLDN are much higher than those in WWLLN, especially during July and over the land, and this is generally true (not shown) that NLDN reported more lightning flashes than WWLLN during warm months over land. The differences are much smaller during cool months and over the coastal regions where NLDN has coverage. Note that the absolute difference in flash count may not necessarily translate proportionally into convective activities in terms of LTA because the LTA technique, as described in Heath et al. (2016), depends on the detection of lightning occurrence (binary “yes” or “no” situation), and not the actual flash count, in a specific time interval at a grid cell.

### 3.2 Precipitation data

The daily precipitation from the Parameter-elevation Regressions on Independent Slopes Model (PRISM)’s high-resolution spatial climate data for the United States (https://climatedataguide.ucar.edu/climate-data/prism-high-resolution-spatial-climate-data-united-states-maxmin-temp-dewpoint, last access: 7 November 2022) is used to evaluate WRF-simulated precipitation over the CONUS, and the NOAA Climate Prediction Center (CPC)’s global unified gauge-based analysis of daily precipitation (https://psl.noaa.gov/data/gridded/data.cpc.globalprecip.html, last access: 7 November 2022) product is employed to assess WRF’s hemispheric precipitation predictions. The daily total PRISM precipitation data are available at 4 km horizontal grid spacing over the CONUS, and the annual CPC precipitation (partitioned into daily totals) is available globally at a 0.5° latitude and × 0.5° longitude grid (720 × 360) resolution. These datasets were regridded to the WRF modeling domains for the 12 km CONUS and the 108 km Northern Hemisphere to pair with model simulations in time and space. To assess the simulated precipitation over the oceans, especially in the tropical regions where no gauge-based measurement is available, products from the Global Precipitation Measurement (GPM; Huffman et al., 2015; Asong et al., 2017), a joint mission co-led by NASA and the Japan Aerospace Exploration Agency (JAXA) and comprised of an international network of satellites that provide the next-generation global observations of rain and snow, are employed. The Integrated Multi-satellitE Retrievals for GPM (IMERG) long-term precipitation data products (https://arthurhouhttps.pps.eosdis.nasa.gov/gpmdata/YYYY/MM/DD/imerg/, last access: 7 November 2022; registration is required for access) cover the entire globe with a 0.1° latitude and × 0.1° longitude grid resolution. To compare with WRF-simulated hemispheric precipitation, the daily mean precipitation data from the IMERG V06 dataset (https://gpm.nasa.gov/data/directory, last access: 7 November 2022) from 2016 is regridded onto the hemispheric WRF domain. The research-quality gridded IMERG V06 dataset Final Run product estimates precipitation using quasi-Lagrangian time interpolation, gauge data, and climatological adjustment.

### 3.3 Ground-based meteorological data

The impacts of user-definable parameter values associated with KF and datasets for LTA were quantified for simulated near-surface meteorological variables such as precipitation, 2 m temperature (*T*2), water vapor mixing ratio, wind speed, and wind direction. The simulated meteorological fields from WRF are compared against observations from NOAA National Centers for Environmental Information (NCEI) land-based stations, which are archived from data collected globally (https://www.ncei.noaa.gov/products/land-based-station, last access: 7 November 2022). The Atmospheric Model Evaluation Tool (AMET; Appel et al., 2011) is used to pair surface observations with model-predicted values in both space (bilinear interpolation) and time (h).

### 3.4 Model configurations and simulation details

The WRF model (Skamarock and Klemp, 2008) version 4.1.1 (WRFv411; https://github.com/wrf-model/WRF/releases/tag/v4.1, last access: 7 November 2022), with LTA updates to Heath et al. (2016; as described in Sect. 2) is used to perform simulations over the CONUS and the hemispheric domains. The CONUS domain is configured with 36 vertical levels and 12 km horizontal grid spacing with 472 × 312 grid points. The hemispheric domain is configured with 45 vertical levels and 108 km horizontal grid spacing, with 200 × 200 grid points, that covers the entire Northern Hemisphere and the northern border of the Southern Hemisphere along the Equator. The simulation period for CONUS simulations is from April to July in 2016, with a 10 d spin-up period from 22 March; for the hemispheric domain, annual simulations for 2016 are performed. Our analysis focuses on July, when convective activities are often the most prevalent over the CONUS; other months are examined in the hemispheric simulations which simulate the year-round convective activities in the tropics. The detailed configurations of cloud microphysics, land surface parameters, radiation schemes, and four-dimensional data assimilation (FDDA) are the same as described in Heath et al. (2016), and the sample WRF name list input files for both the CONUS and hemispheric simulations are included in the Supplement (Tables S1 and S2). Data assimilation in the form of FDDA more specifically follows Heath et al. (2016), with updates noted in Gilliam et al. (2021), for the hemispheric domain where ~ 28 km National Centers for Environmental Prediction (NCEP) Global Forecast System (GFS) analyses were used to nudge tropospheric temperature, moisture, and wind above the planetary boundary layer. For the CONUS domain, the same nudging was applied, but 12 km North American Mesoscale (NAM) model analyses were leveraged. These two analysis datasets are a blend of a short-term forecast with a comprehensive set of surface, upper-air, radar, aircraft, satellite, and other observations, like sea surface temperature, that represent the best estimate of the state of the atmosphere at any given time.

The KF scheme includes two options to trigger convective activity. Trigger 1 is based on a mass-conservative cloud model, which includes parameterized moist downdrafts, entrainment, and detrainment at the cloud edge (Kain and Fritsch, 1990, 1993) and allows interaction between cloud and environment, and it is the default option for most applications. Trigger 2 is an alternate option based on Ma and Tan (2009) and is a moisture-advection-modulated trigger function to improve results in subtropical regions when large-scale forcing is weak. In addition, the KF scheme is called by default at every time step, but it can be configured to only update convective parameters on a user-definable time increment. In this study, sensitivities are conducted with the version of the KF trigger (i.e., Trig1 and Trig2; abbreviated as K1 and K2 in Table 1, respectively) and the frequency at which KF is called (i.e., cudt). Two sensitivities on cudt were performed, i.e., one where KF is called at each model integration time step (i.e., “Cudt0”; abbreviated as C0 in Table 1), and the other where KF is updated after every 10 min of integration time (i.e., “Cudt10”; abbreviated as C10 in Table 1). The time step is 1 min (Table S1) and 3 min (Table S2) for the CONUS and hemispheric WRF simulations, respectively. The sensitivities to KF trigger and update frequency are combined in a matrix of simulations that also are conducted with/without LTA, and they are listed in Table 1. All eight simulations are performed for both the CONUS and the hemispheric domains. For LTA cases, lightning flashes from both NLDN and WWLLN are used over the CONUS domain, and lightning flashes from WWLLN are used for the hemispheric domain. For convenience of description, the cases without LTA are collectively referred to as base cases, and the cases with LTA are referred to as LTA cases. To further distinguish the lightning networks, the LTA cases are also referred to as LTA NLDN (or simply NLDN) and LTA WWLLN (or simply WWLLN) cases, respectively.

### 3.5 Evaluation methodologies

**Figure 2.**
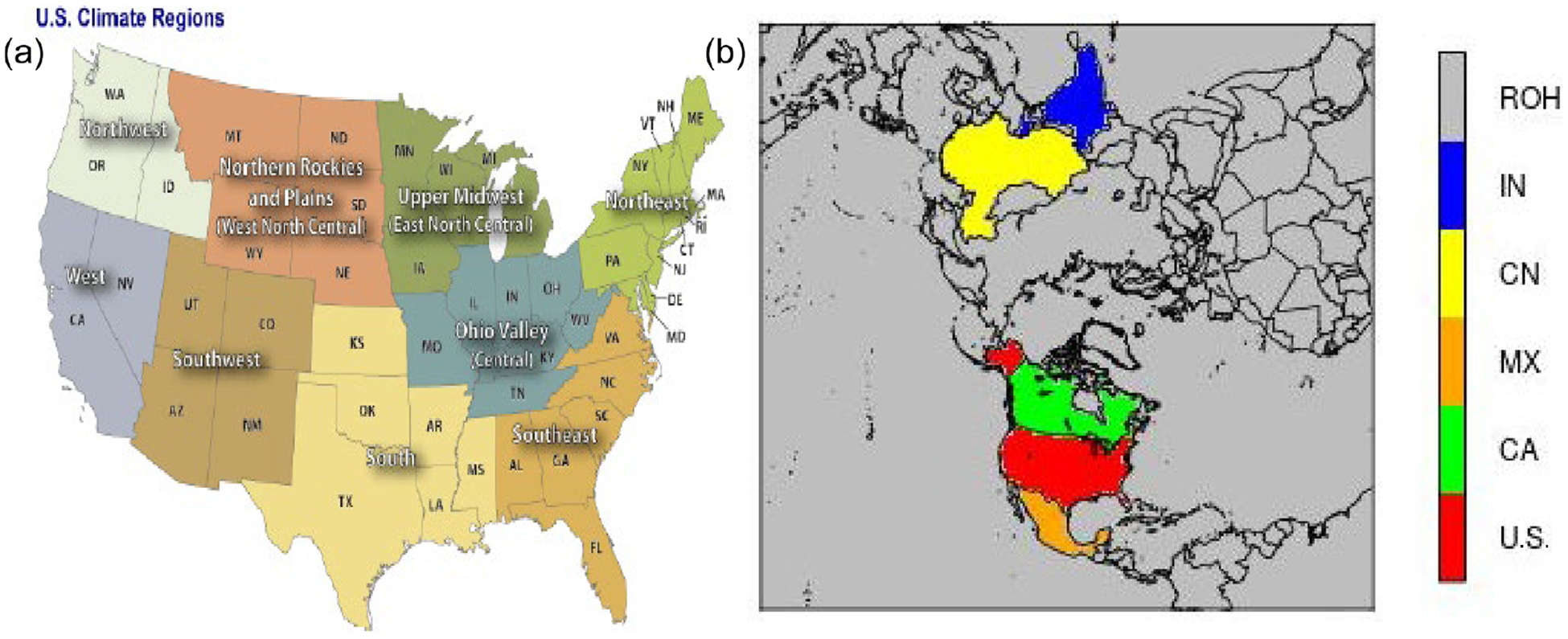
Analysis regions (countries), **(a)** the climate regions in the CONUS, and **(b)** the countries over the Northern Hemisphere, with U.S. for the United States, CA for Canada, MX for Mexico, CN for China, IN for India, and ROH for other countries/regions, except the five specific countries in the hemispheric domain. The U.S. climate regions are northeast (NE), southeast (SE), Ohio Valley central (OVC), upper midwest (UM), south, west north central (WNC), southwest (SW), northwest (NW), and west.

The assessment of the impact of LTA on model performance is focused on precipitation, since that is the most-affected variable, though other near-surface variables are also evaluated. Due to the highly heterogeneous nature of thunderstorms and lightning over space, in addition to examining the overall statistics across the modeling domain, statistics are analyzed to assess the impact of LTA over U.S. climate regions (https://www.ncei.noaa.gov/monitoring-references/, last access: 7 November 2022) in both domains and some of the larger countries in the hemispheric simulations. Figure 2 shows these climate regions over the CONUS modeling domain and the selected countries (also referred to as regions) in the hemispheric modeling domain.

The statistical metrics in this analysis include the widely used correlation coefficient (*r*) to measure the linear association of measured and simulated variables, mean bias (MB), and normalized mean bias (NMB), to quantify the departure of simulated values from measured values, and the root mean square error (RMSE) and normalized mean error (NME), to elucidate the errors associated with model simulations. More emphasis is placed on certain metrics rather than others, depending on the nature of the simulated quantity. For instance, with precipitation, the correlation coefficient (if the model can simulate rainfall at the right time and location) and MB and NMB (if the model over- or underestimates rainfall amount) are more straightforward than the error metrics (though they are still relevant), but MB and NMB are inappropriate to evaluate wind directions.

## 4 CONUS WRF simulations

As shown in Table 1, four base (without LTA) cases, four LTA cases using lightning flash data from NLDN, and four LTA cases using lightning flash data from WWLLN over the CONUS domain were performed using the combinations of two trigger options and two convective update (cudt) intervals, respectively. For the LTA cases, when lightning flashes were not present, the ShallowOnly option (Heath et al., 2016) was used (Table S1).

### 4.1 Precipitation

**Figure 3.**
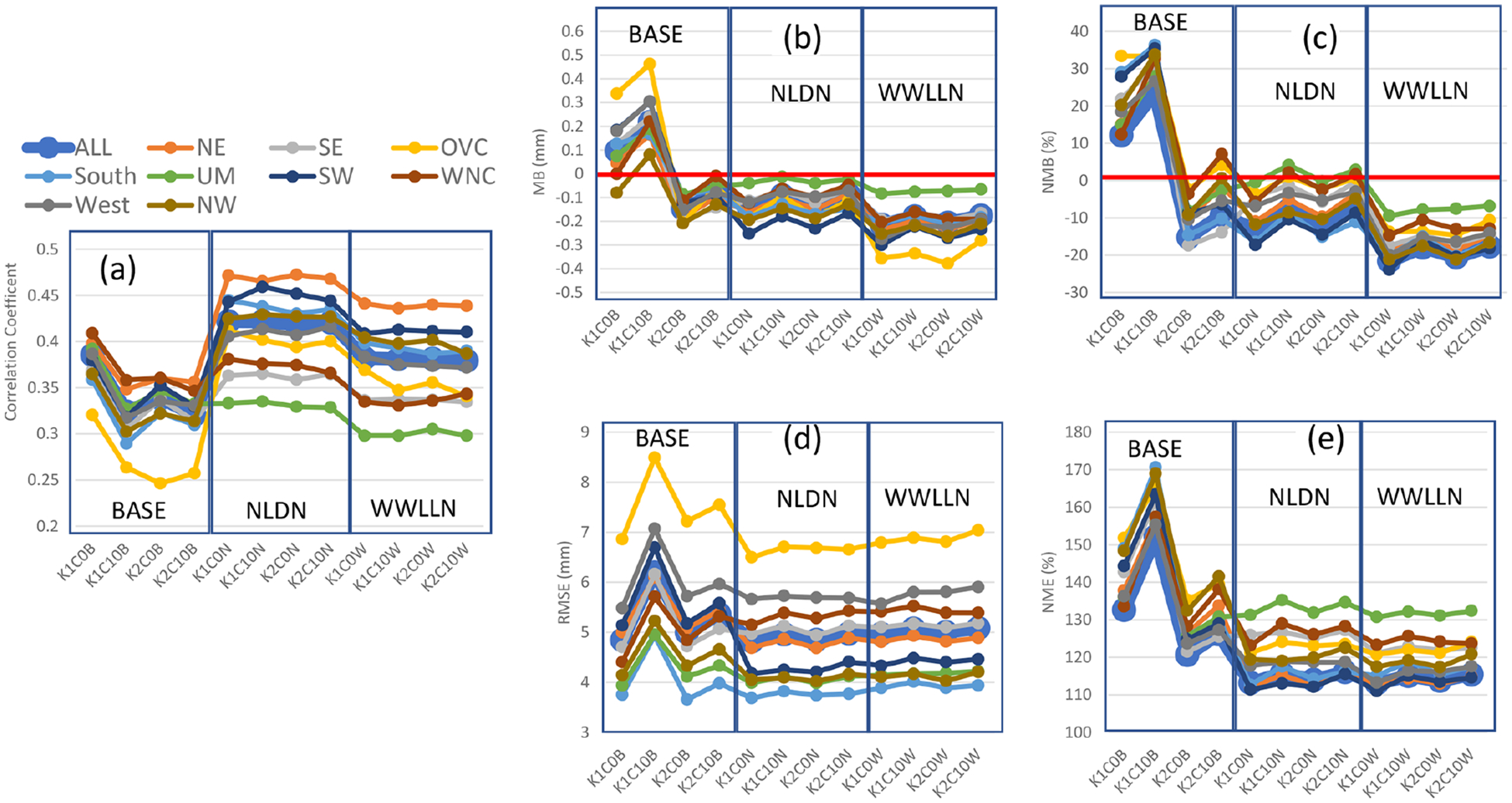
Monthly mean statistics for precipitation from base and LTA simulations comparing to the values from PRISM for the modeling domain and the climatological regions over the CONUS, respectively, during July 2016. **(a)** Correlation coefficient, **(b)** MB, **(c)** NMB, **(d)** RMSE, and **(e)** NME. In each plot, there are three sets of simulations (base, LTA with NLDN, and LTA with WWLLN), and each set has four cases from the combinations of cumulus parameters.

Figure 3 displays the July 2016 mean statistics generated by pairing the gridded WRF precipitation with the values from PRISM in time and space for each of the U.S. climatological regions. As shown in Fig. 3, the base simulations present the more dramatic fluctuations among cumulus parameter sensitivities than the LTA cases. With Trig1, when the cudt is changed from 0 to 10, the correlation coefficient is substantially reduced across all the regions (Fig. 3a), and increases in biases (overestimate of precipitation; Fig. 3b and c) and errors (Fig. 3d and e) are also worsened by less frequent cumulus updates. With Trig2, the biases (MB and NMB) changed from overestimation to underestimation, and the errors (RMSE and NME) were smaller compared to Trig1. Though the setting for cudt altered simulations with Trig2, the difference was smaller than the cases with Trig1. In general, the Trig1 cases tended to produce more precipitation (overestimate compared to PRISM precipitation) than the Trig2 cases (underestimate compared to PRISM precipitation), and the Cudt10 cases generated more precipitation than the Cudt0 cases. Among the four cases in the base model simulations, the K1C0 case (Trig1; Cudt0) is the most favorable in terms of the correlation coefficients and precipitation biases, but the error statistics, especially NME, may not be the most desirable.

Using LTA (Fig. 3), the correlation coefficients significantly increased over the domain and across the regions (from the range of ~ 0.25 to ~ 0.40 to the range of ~ 0.30 to ~ 0.48), relative to the base cases. Though the LTA WWLLN cases had lower correlation compared to the LTA NLDN cases due to the lower detection efficiency of lightning flashes in WWLLN, the improvement was still rather considerable compared to the base cases. The biases in the LTA NLDN cases are most favorable with values that are negative but closest to zero (small underestimate). The LTA WWLLN cases produced larger negative biases than the base cases and LTA NLDN cases, which is, again, related to detection efficiency of the networks. All the LTA cases (both NLDN and WWLLN) produced smaller errors than the base cases, and the differences between the NLDN cases and WWLLN cases were minimal. Comparing the LTA cases with the base cases, one noticeable feature is that, with the different trigger and cudt values, all the statistics fluctuated dramatically from one case to another in the base cases, but fluctuation among the LTA cases was minimized and negligible. This is expected, as the moisture and temperature perturbations used to trigger convection with LTA (Sect. 2) will take precedence over the trigger options, and grouping the lightning data into 30 min bins should mitigate the influence of the cudt option. These features were deliberately incorporated into the LTA technique for precisely these reasons, but this paper documents their systematic testing.

Examination of the statistics across the climatological regions over the CONUS domain indicates that the Ohio Valley (OVC) stands out among all the regions with the lowest correlation coefficients and largest RMSE values in all the base cases. However, with LTA, the correlation coefficients in OVC were brought to the median range among other regions, though the RMSE values were still the largest in that region; these features in OVC are more understandable, as manifested in Fig. 12 and examined in detail in Sect. 5. Other statistics in OVC with LTA were comparable with other regions, except for relatively larger negative MB values associated with the LTA WWLLN cases. Another obvious characteristic with regards to correlation coefficients and errors (RMSE and NME) was that there was more spread among the regions in the LTA cases than in the base cases (except in OVC), which resulted from the geographically heterogeneous nature of convective precipitation and the associated observed lightning intensity across the regions.

To alleviate the underestimation of precipitation in the LTA WWLLN cases, additional simulations (K1C10Ws0 and K2C10Ws0, where K1C10W and K2C10W are the same as in Table 1, while s0 means zero suppression when lightning flash is not present) were performed by switching the suppression option, as described in Heath et al. (2016), from ShallowOnly to “NoSuppress.” This modification still triggers deep convection where lightning is observed; however, at grid points without lightning, the KF scheme is configured to run normally (i.e., the same as in the base cases). As shown in Fig. S1 in the Supplement, the correlation coefficients in the WWLLN+s0 cases were comparable with other LTA cases, and the values in the K2C10Ws0 case were similar to the NLDN cases and improved upon the K1C10W case. The MB in the WWLLN+s0 cases were mostly positive (overestimate), which is expected because the KF scheme has more freedom to activate deep convection. The K2C10Ws0 case produced the most desirable results (domain mean MB is nearly zero) among all the cases. However, the biases associated with LTA simulations using the NoSuppress option are affected by both the lightning detection efficiency and the domain resolutions, which is more evident in the LTA simulations over the hemispheric domain in Sect. 5.

### 4.2 Other near-surface meteorological variables

**Figure 4.**
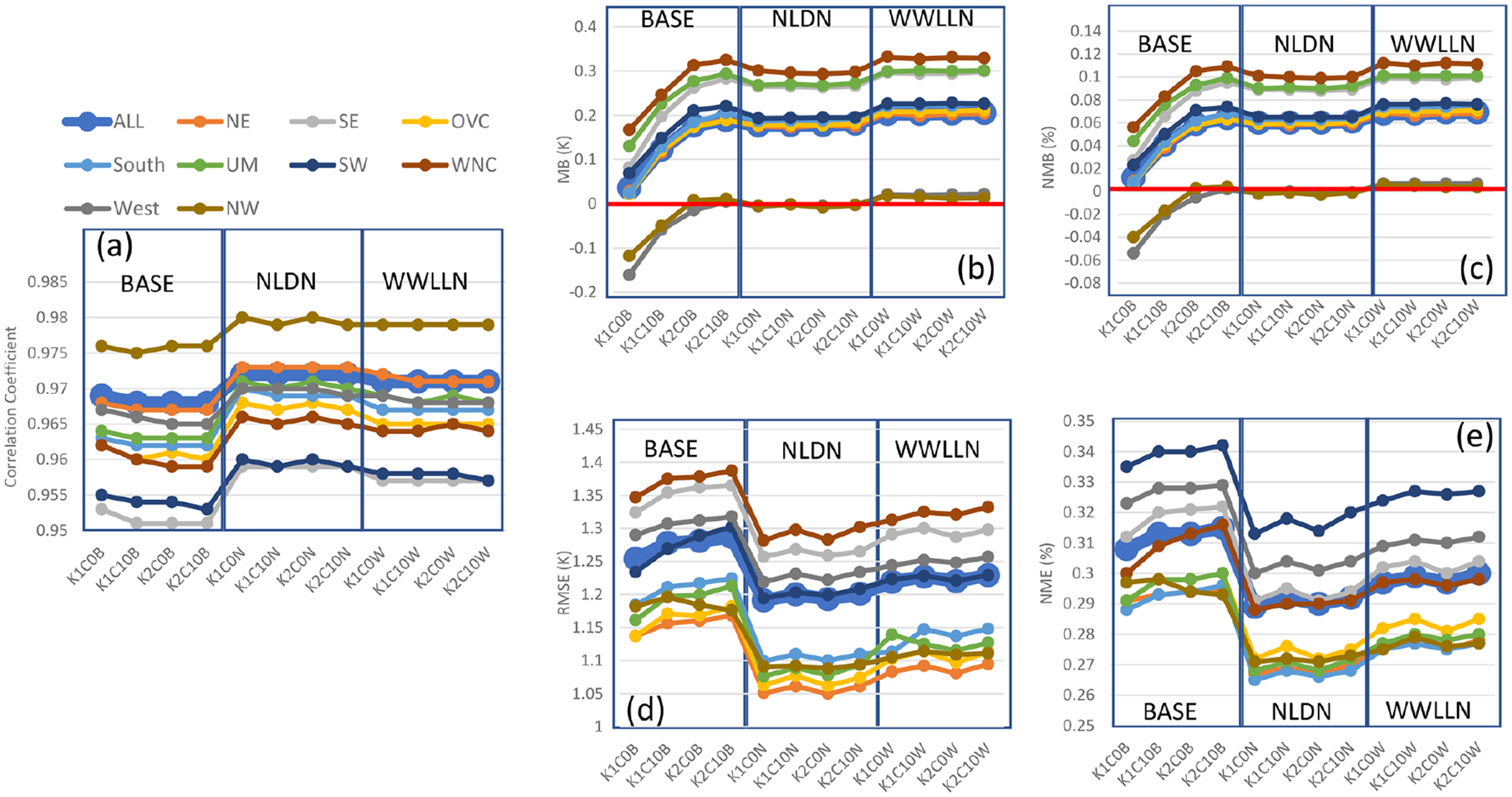
Same as Fig. 3 but for 2 m temperature (*T* 2) in that the simulated *T* 2 values are paired with observations from NCEI’s land-based stations in time and space (hourly mean values).

Besides precipitation, *T* 2, water vapor mixing ratio, wind speed, and wind direction are also analyzed. As shown in Fig. 4, *T* 2 in the base cases has correlation coefficients over the CONUS domain and all the regions ranging from ~ 0.95 to 0.98. With LTA, the correlations for *T* 2 were further improved for all the regions, with WWLLN cases performing slightly worse than the NLDN cases. The impact of cumulus parameters on correlations was minimal for the base and LTA cases. However, the cumulus parameters seem to impact the biases (MB and NMB; Fig. 4b and c) and errors (RMSE and NME; Fig. 4d and e) in the base cases across all the regions, and like precipitation, all the LTA cases minimized the impact of different cumulus parameter values. All the LTA cases reduced the errors (RMSE and NME) associated with *T* 2 across all the regions, with NLDN slightly better than WWLLN. In summary, the *T* 2 statistics were improved by using LTA, and the WWLLN cases were comparable to the NLDN cases with a slight degradation for all the regions.

**Figure 5.**
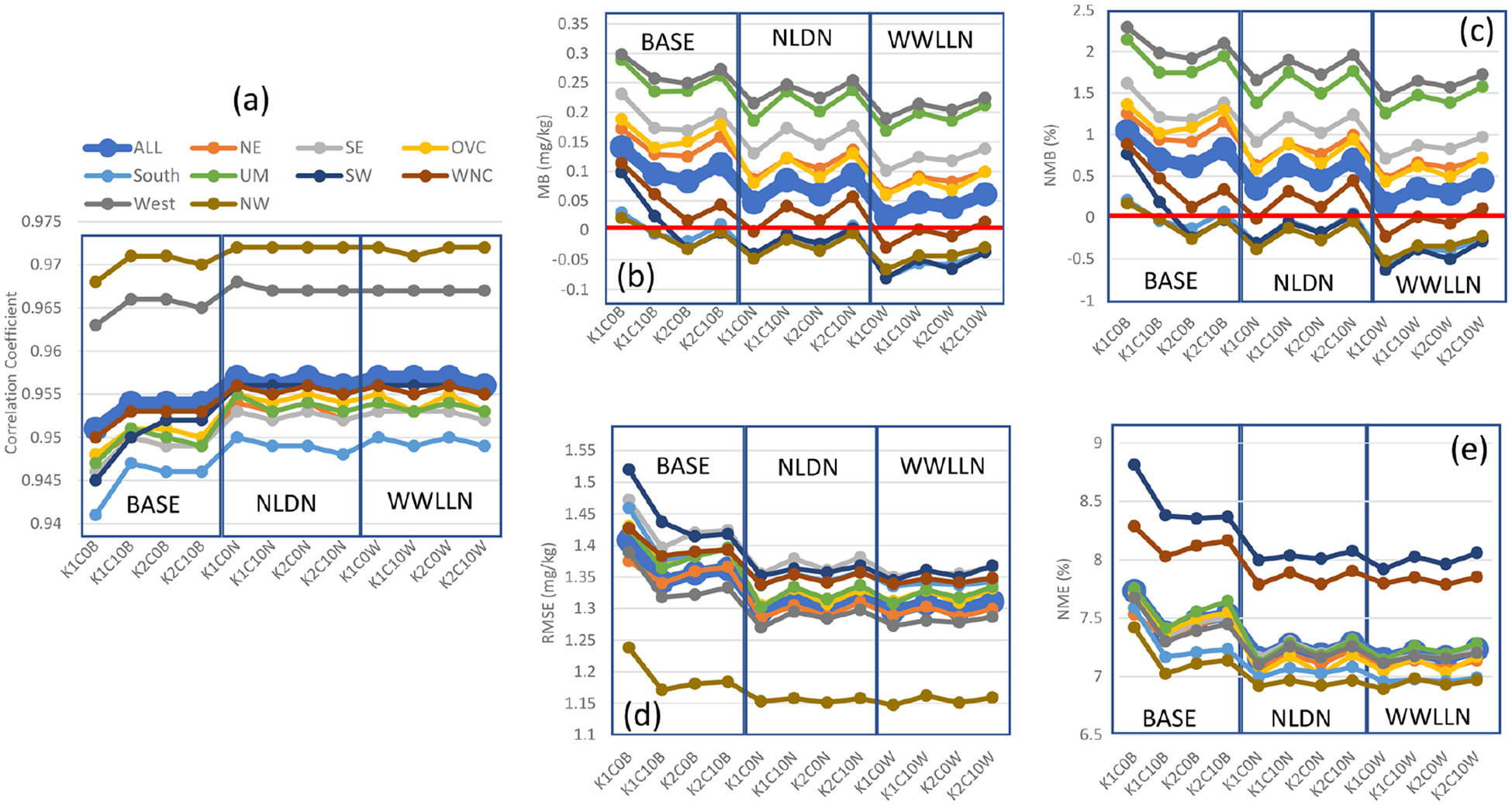
Same as Fig. 4 but for 2 m water vapor mixing ratio.

The 2 m water vapor mixing ratios metrics (Fig. 5) of the cases, in general, resemble those of *T* 2, in that the LTA cases have slightly increased the correlation coefficients from the already well-simulated base cases. More spread occurs for biases (MB and NMB; Fig. 5b and c) and within the base cases for errors (RMSE and NME; Fig. 5d and e). Regional spread in these statistics is attributed to the diverse air mass types that drive large differences in the moisture content and convective activity. Even though the values were low for both errors and biases (< 0.5 %), using either LTA technique is an improvement over the base cases.

**Figure 6.**
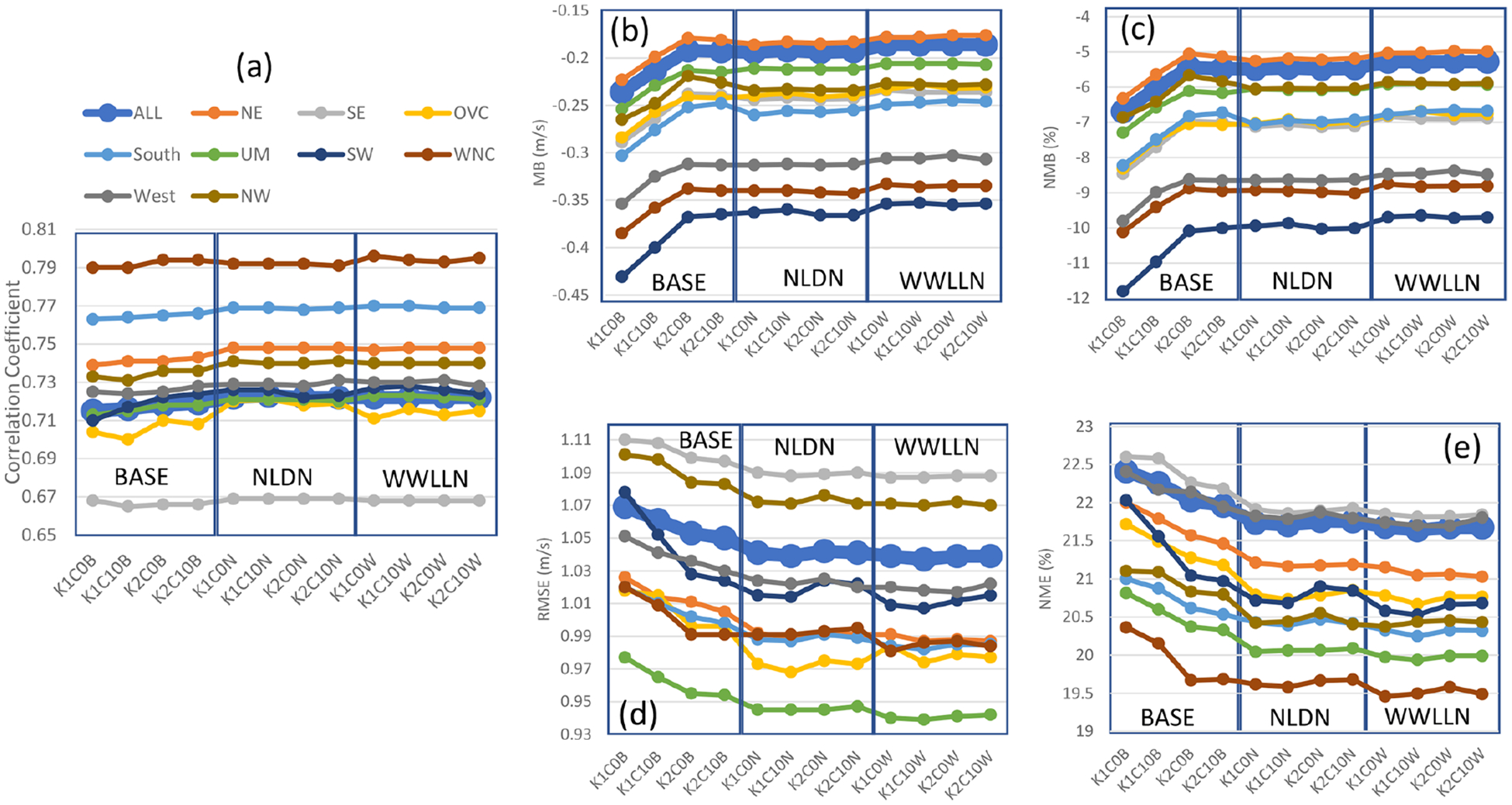
Same as Fig. 4 but for 10 m wind speed.

The cumulus parameters and LTA showed less of an impact on the correlations for 10 m wind speed, but the impacts on biases and errors were noticeable (Fig. 6). All the model cases underestimate wind speed (~ 5 %–12 %, depending on regions and model cases), and the cumulus parameters caused relatively large differences in the metrics of the base cases with both trigger and cudt options contributing the most. Overall, using Trig2 with Cudt10 is most favorable in terms of biases (less underestimate) and errors (smaller errors) among the base cases. In all the LTA cases, the underestimation was reduced when compared to the base cases, and errors were reduced with negligible differences among the cases with different cumulus parameters and assimilating lightning data from the different networks. Similar behavior was observed for wind direction where only the correlation coefficient, MB, and RMSE are displayed in Fig. S2 in the Supplement because normalized metrics do not apply.

## 5 Northern hemispheric WRF simulations

As shown in Table 1, the model cases performed over the Northern Hemisphere are similar to those performed over the CONUS, but with LTA cases using lightning data from WWLLN that was gridded on the domain with 108 km horizontal grid spacing.

### 5.1 Precipitation

**Figure 7.**
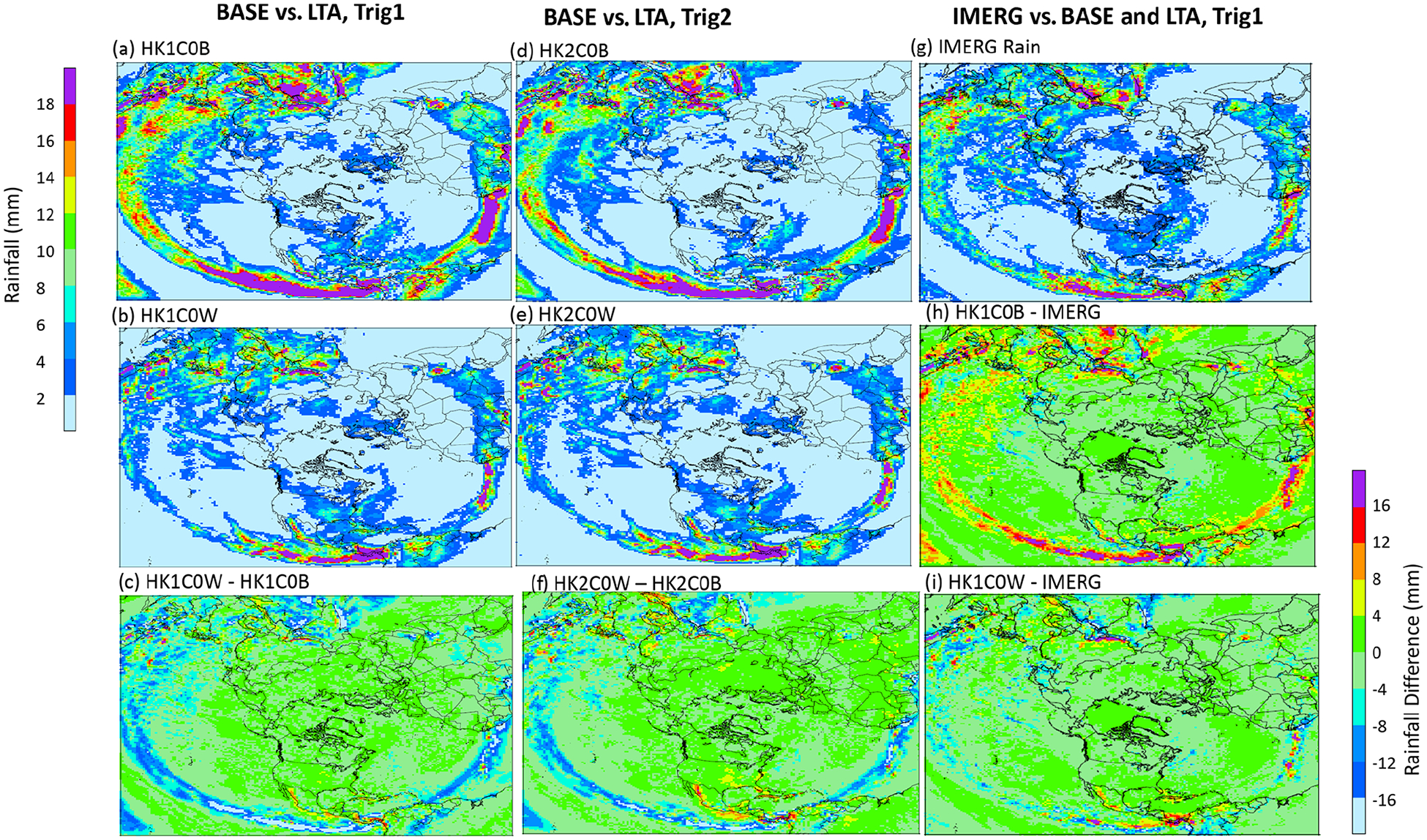
The mean daily rainfall during July 2016, simulated by base model cases (panel **(a)** HK1C0B and panel **(d)** HK2C0B), LTA cases (panel **(b)** HK1C0W and panel **(e)** HK2C0W), and the satellite GPM-produced rainfall **(g)** and the differences between the LTA and base cases (panel **(c)** HK1C0W–HK1C0B and panel **(f)** HK2C0W–HK2C0B) and between the simulated cases and satellite IMERG products (panel **(h)** HK1C0B–IMERG and panel **(i)** HK1C0W–IMERG), are shown. Note that the left legend applies to the rain maps **(a, b, d, e, g)**, and the right legend applies to the difference plots **(c, f, h, i)**.

**Figure 8.**
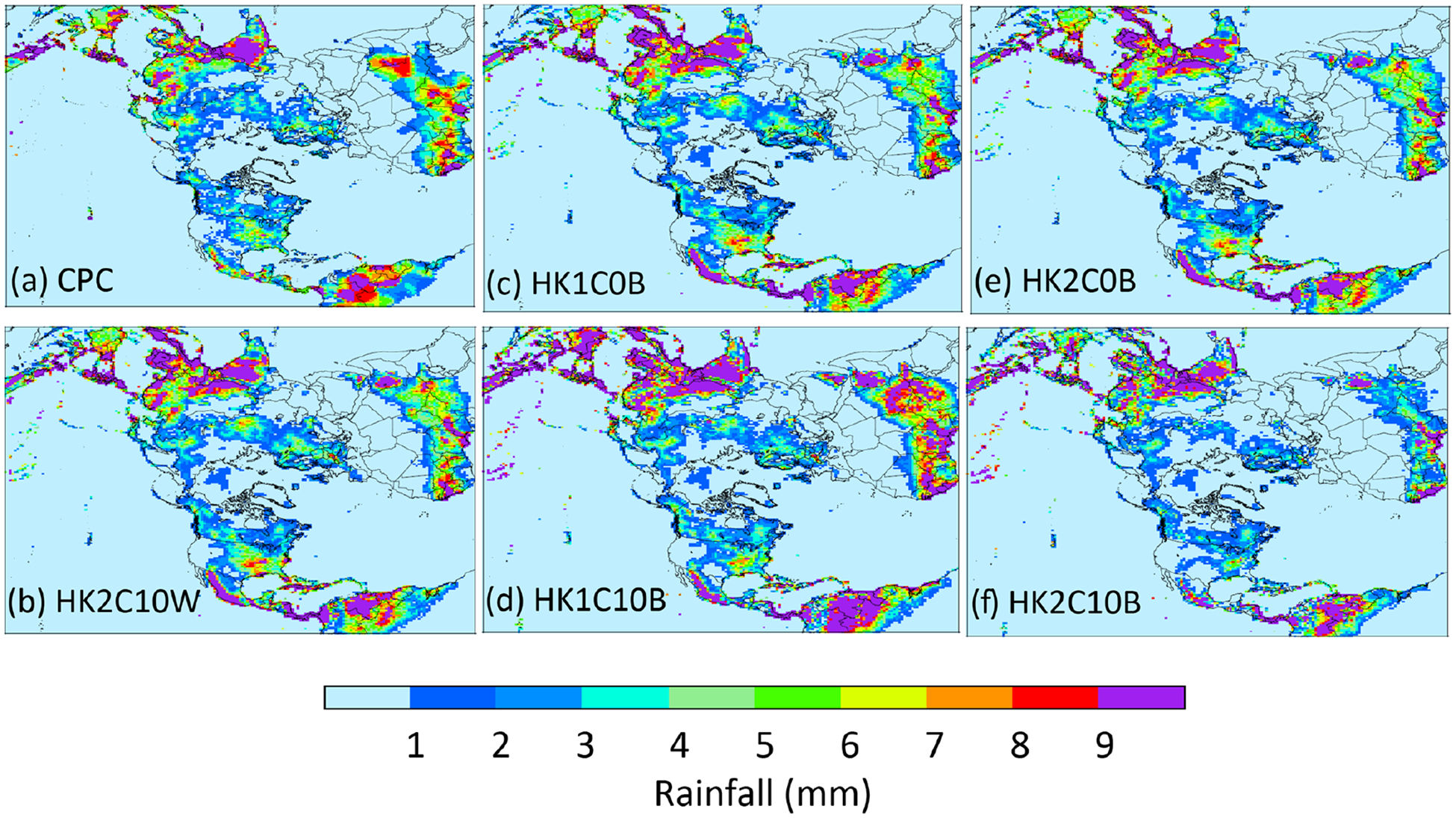
CPC rainfall **(a)** and simulated **(b–f)** mean daily precipitation during July 2016 over the hemispheric domain. The LTA configuration is represented by one case (panel **(b)** HK2C10W), since all the LTA cases with different cumulus parameters produced similar results. All base cases are shown here **(c–f)** because the cumulus parameters do impact the simulated precipitation when not using LTA.

Before comparing the simulated precipitation with available observations, the examination begins with how the WRF-simulated precipitation with and without LTA compares spatially over the Northern Hemisphere. Figure 7 displays the mean daily precipitation during July 2016 from two LTA cases and two base cases (Trig1 and Trig2) and the corresponding differences between LTA and base (LTA – base) cases with the same trigger values, and Fig. S3 in the Supplement presents the mean daily precipitation differences between HK1C0W and HK1C0B cases throughout 2016. Compared to the base cases, the LTA cases produced significantly less rainfall along the equatorial regions but generally more rainfall away from the Equator, especially over the midlatitude land regions. Because no gauge-based observational data are available over the ocean, the IMERG precipitation for July 2016 is presented in Fig. 7g, with the difference plots from the base case (HK1C0B) and the LTA case (HK1C0W) being displayed in Fig. 7h and i, respectively. Over the equatorial regions, the precipitation simulated by the LTA cases (Fig. 7b and e) more closely resembled the IMERG precipitation than the base cases. The difference plots clearly indicate that the base cases significantly overestimated, and the LTA cases slightly underestimated, the precipitation over large areas in the equatorial regions. Similar results persisted throughout the year, as shown in Figs. S4 (the difference in mean daily precipitation by month between the base case, HK1C0B, and the IMERG product) and S5 (the difference in mean daily precipitation by month between the LTA case, HK1COW, and the IMERG product). Next, the WRF-simulated precipitation is compared with the CPC gauge-based analysis values over land. Figure 8 displays the CPC rainfall and simulated mean daily precipitation during July 2016, along with the estimates from the LTA and base cases with different cumulus parameters. Since the gauge-based observational values are only available over land, the simulated values in Fig. 8 are only displayed over land. As shown in Fig. 8, all the model cases simulated the overall spatial pattern of higher values in the tropical regions and lower values in high-latitude regions. However, subtle differences existed from case to case in different regions. For example, the HK1C10B case (Fig. 8d) and the HK2C10B case (Fig. 8f) produced the highest and the lowest precipitation over Africa and South America (along the Mexico coast to the South American continent) within the modeling domain.

**Figure 9.**
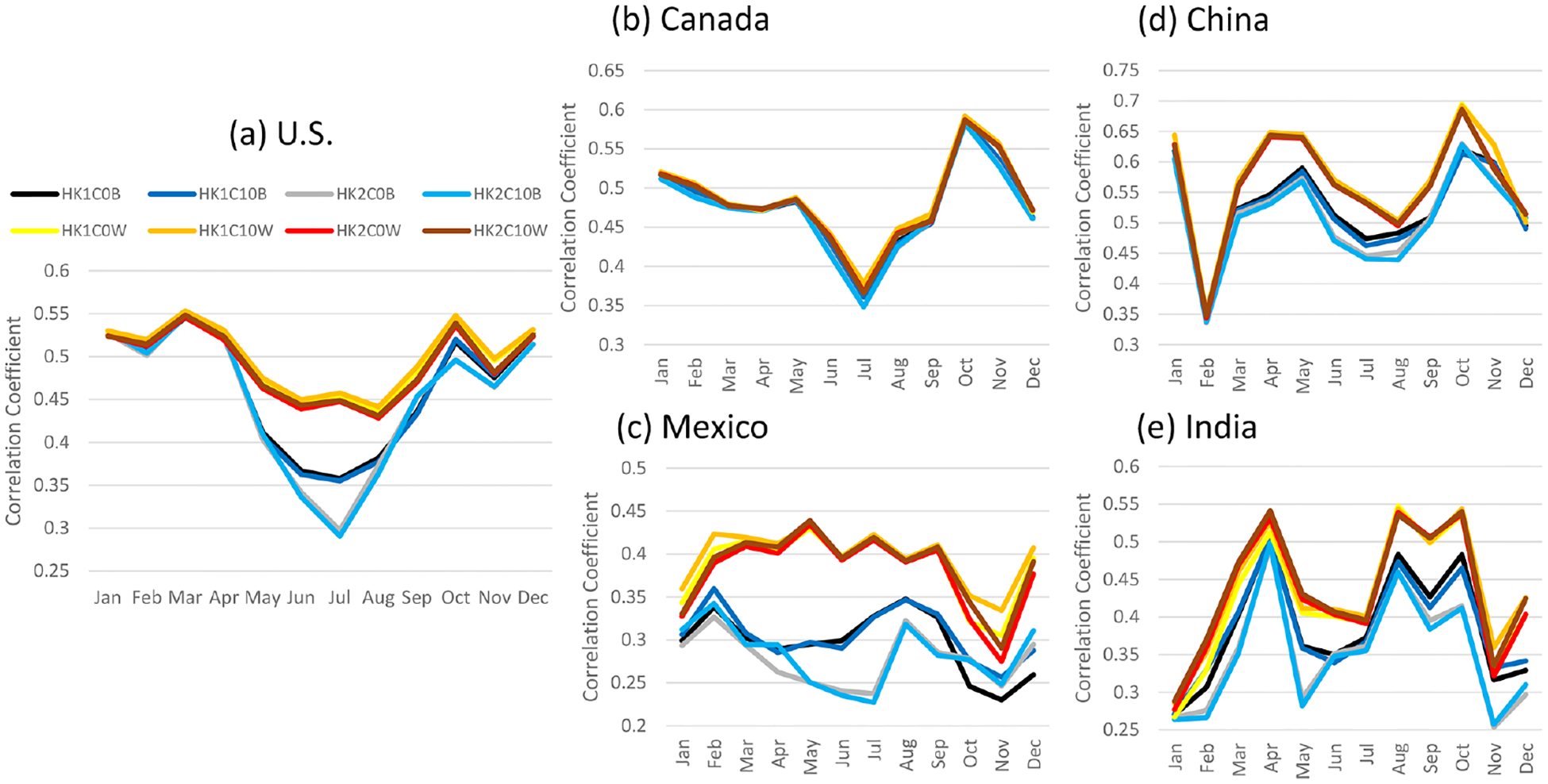
The monthly correlation coefficient between CPC and simulated precipitation in selected countries, i.e., **(a)** the United States, **(b)** Canada, **(c)** Mexico, **(d)** China, and **(e)** India. Note that all the base cases are plotted in cool colors and LTA cases in warm colors.

All the LTA cases uniformly produced larger correlation coefficients than the base cases (Fig. 9) when and where convective activities were prevalent. In the U.S., convective activities occur during warm months (from May to September), while in Mexico and India, convection is active throughout the year. In Canada, convective activities are less frequent because of the cooler temperatures and low moisture at the high latitude. When and where convection was active, the cumulus parameters produced significant differences in modeled convective activity, as correlation coefficients are higher in the base cases with Trig1. Similar to the simulations over the CONUS domain, the cumulus parameters had a minor impact on the correlation coefficients for the LTA cases regardless of the regions. This indicates that, even with the less dense WWLLN lightning observations, using LTA improves the timing and location of deep convection.

**Figure 10.**
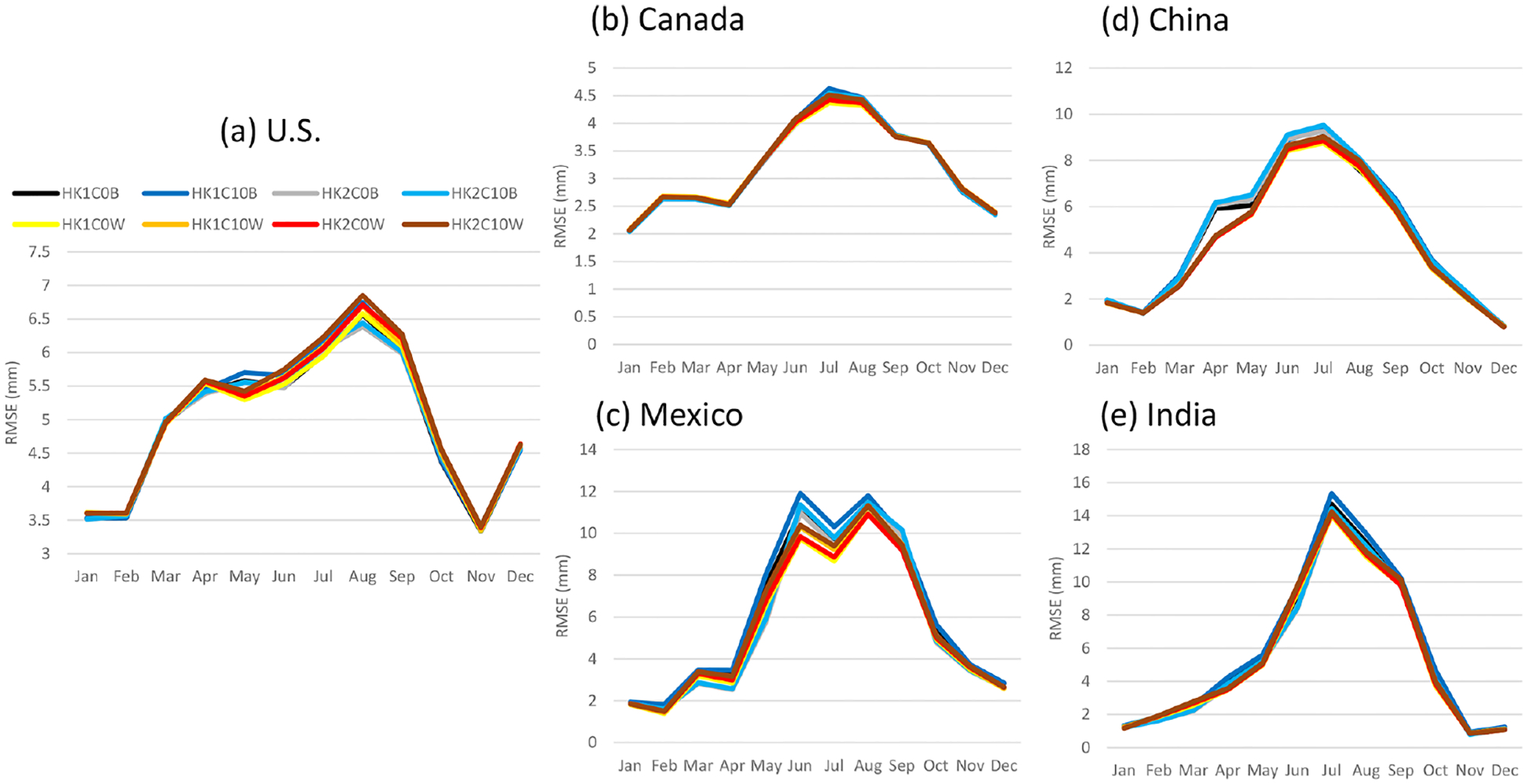
Same as Fig. 9 but for RMSE.

**Figure 11.**
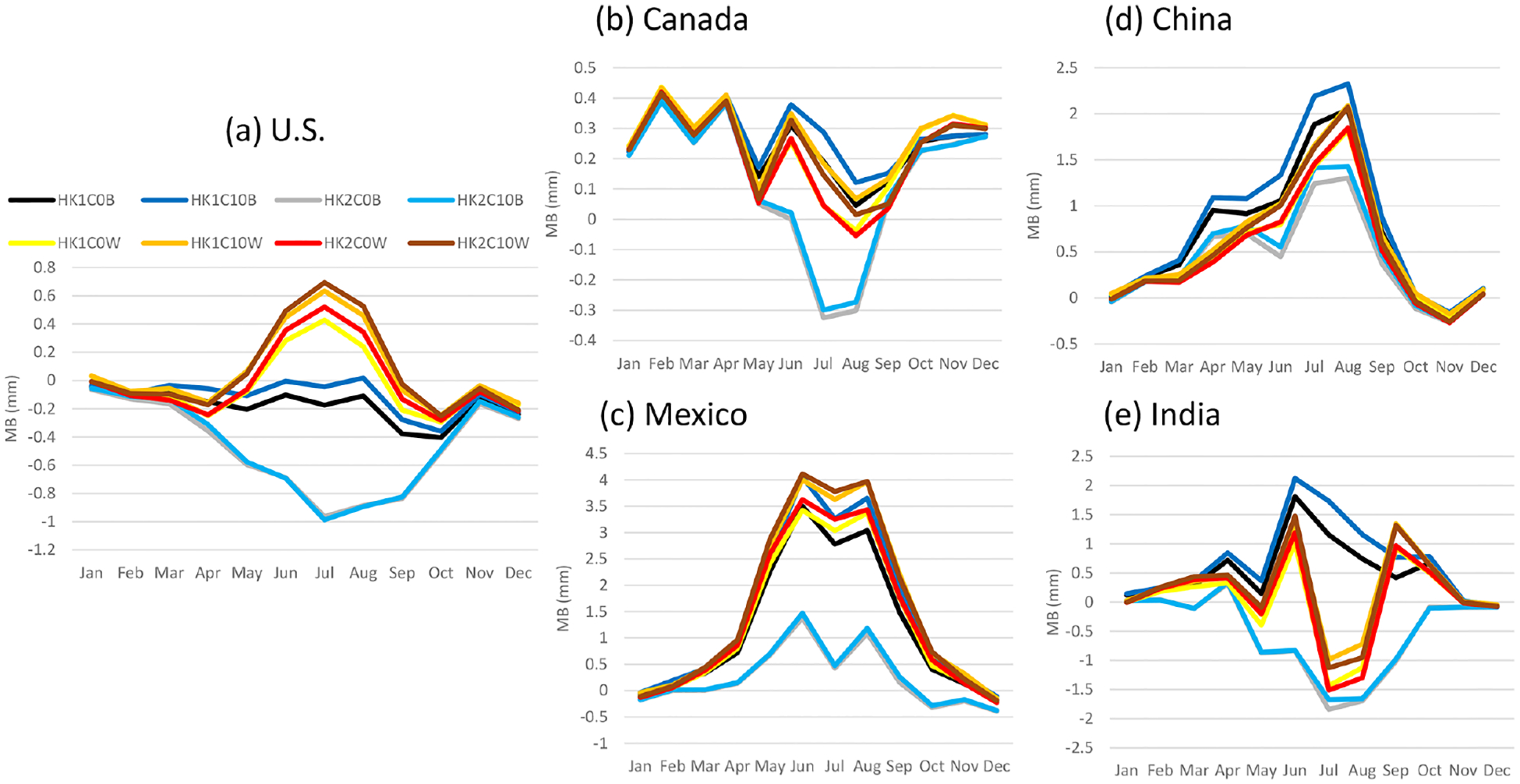
Same as Fig. 9 but for MB.

RMSE were comparable for all the model cases across the selected regions (Fig. 10), with the LTA cases pointing to lower values than the base cases at all the regions except for the U.S., where the LTA and base cases alternated to have slightly lower RMSE values over each other during the year. Alternatively, the MB values varied significantly among the model cases and across the regions, as shown in Fig. 11. One common feature is that the differences among the LTA cases were small, but two distinctly separate groups among the base cases were noted in all the regions; the cases with Trig1 had always significantly greater precipitation values than the cases with Trig2. In China and Mexico, all the simulations overestimated the precipitation through the year, except for a small underestimation during the cool months (October–December). In India, the overestimates and underestimates were equally split among the model cases, with dramatic changes from month to month in the same model case. The behavior of MB values among the model cases and through the year was more stable for the U.S. (to a lesser extent in Canada) than in other regions for which the base cases with Trig1 have the best performance (MB values near zero), the base cases with Trig2 significantly underestimated precipitation over land during convective season, and all the LTA cases overestimated precipitation over land during the warm months. Here we offer two plausible explanations for the drastically different behaviors of the MB values associated with precipitation in different regions.

First, from the modeling point of view, the WRF model is widely studied and applied in North America, especially in the U.S. As a result, more accurate observation-based datasets are available to nudge WRF through FDDA (Liu et al., 2008), and all the work has led to the best performance over the U.S. for the recommended default set of the convective trigger and update frequency for the cumulus scheme. Second, from the observational point of view, the CPC rainfall dataset is built upon field gauge measurements that may vary in accuracy and consistency from country to country. As shown in Fig. S6 in the Supplement, the NMB values were generally in the range of −50 % to 50 % in the U.S. and Canada (comparable to the NMB values for the 12 km CONUS simulations against PRISM precipitation, as shown in Fig. 3c), but in other countries, especially during cool months, the values were up to hundreds or even thousands of percent, suggesting few possible observations available in the denominator in NMB calculations. For instance, the highest NMB value in China coincided with the spring festival that is often a long holiday for China, suggesting possible gaps in data collection.

We next focus on the high MB values associated with the LTA cases in the U.S. Consistent in the analysis in Fig. 3b, the LTA WWLLN cases over the 12 km CONUS domain always had larger negative MB (underestimates) than the LTA NLDN cases due to the lower detection efficiency of lightning flashes in WWLLN than in NLDN. However, in the 108 km hemispheric simulations, the same WWLLN datasets produced large positive MB (overestimates) for precipitation. To understand this phenomenon, we need to first examine how the LTA method works. Because it uses a yes/no lightning indicator to trigger convection, the 108 km grid spacing might be too coarse for such a simplistic approach to work. For example, one lightning strike within a 108 km grid cell will trigger deep convection, which, because of the large spatial coverage of the grid cell, can contribute to the high bias in precipitation because convective rainfall is realistically more localized. Although the KF scheme sets a fixed radius for thunderstorms (e.g., Eq. 6 in Kain, 2004), applying the resulting rain over the entire 108km × 108 km grid box could partially explain the excess rainfall. This may also be explained by the fact that the convective timescale formulation in KF scheme was originally developed at grid lengths of 20–25 km (Sims et al., 2017). A potential developmental pathway for the LTA method at these scales is to test different thresholds of the 30 min flash density to ensure sufficient lightning is present to trigger deep convection. Overall, compared to the CPC rainfall, the LTA technique significantly improved the temporal and spatial correlation of convective precipitation, but the precipitation amount was overestimated over the U.S. and other regions for the 108 km modeling domain.

**Figure 12.**
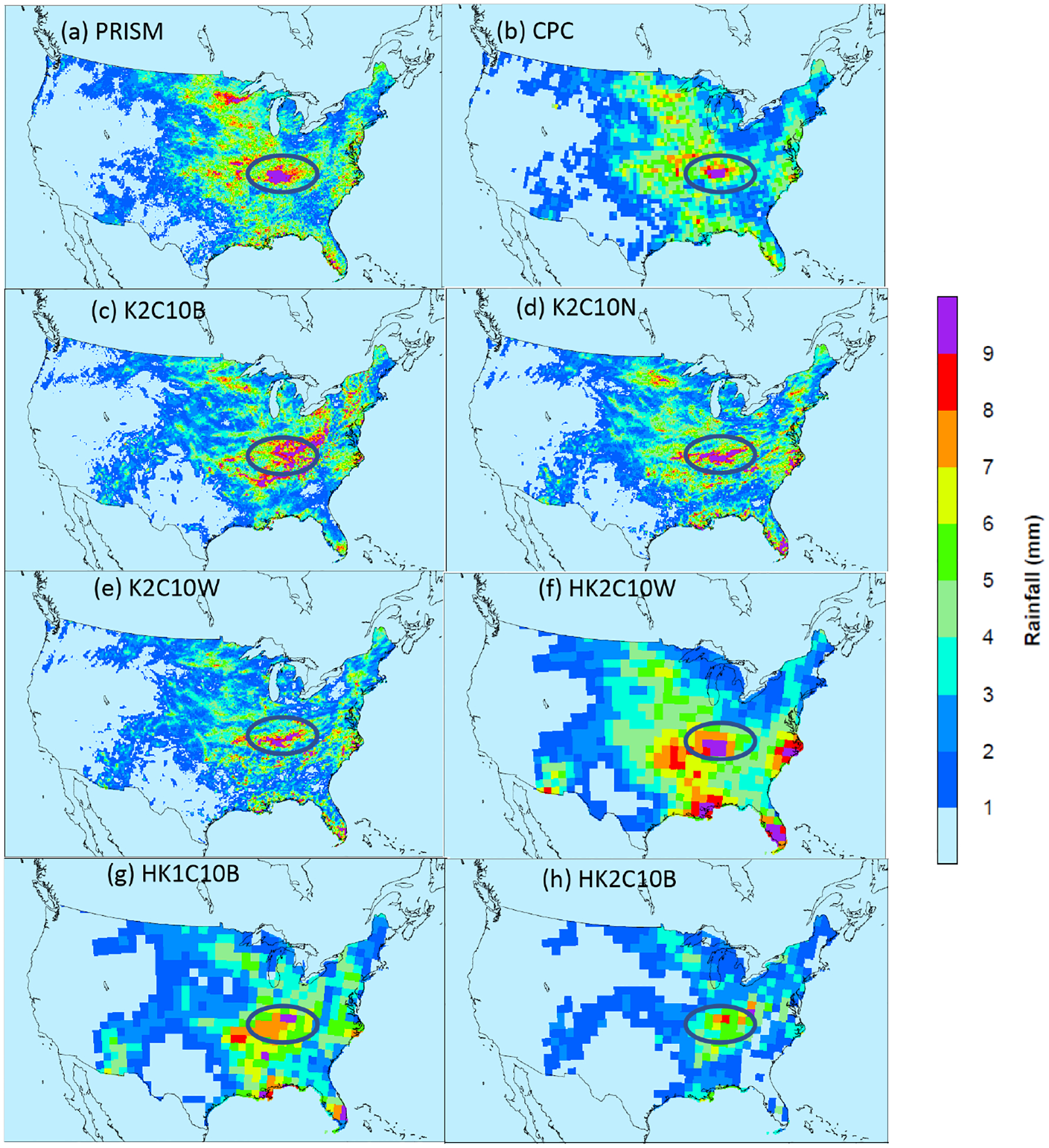
Mean daily precipitation over the CONUS during July 2016 from **(a)** PRISM, **(b)** CPC, **(c)** K2C10B, **(d)** K2C10N, **(e)** K2C10W, and **(f)** HK2C10W, **(g)** HK1C10B, and **(h)** HK2C10B. Note that all the observational-based products and the 108 km hemispheric simulations are regridded onto the 12 km CONUS domain.

To further examine the impact of modeling domain resolutions on convective precipitation, Fig. 12 displays the spatial precipitation from PRISM, CPC (regridded onto the 12 km CONUS domain), and simulated precipitation from one base case and two LTA cases with NLDN and WWLLN data, respectively, over the 12 km CONUS domain and one LTA case over the 108 km hemispheric domain that has been regridded to the 12 km CONUS domain. As shown in Fig. 12a and b, the two observation-based precipitation products, PRISM and CPC, compared well to each other, noting that the PRISM product displays more subtle granularity than the CPC product due to the large difference in spatial resolutions (4 km for PRISM versus 0.5° for CPC). The overall spatial pattern of mean daily precipitation was captured by both the 12 km LTA simulations (Fig. 12d and e), and the 108 km LTA simulation (Fig. 12f). The heaviest rainfall was centered in the OVC area in the observation-based and simulated precipitation maps, but the shape and spread of the rain band were different. The rain band in the 12 km base case (Fig. 12c) was more spread and scattered, with a southwest-to-northeast orientation, while the observation-based products and the LTA cases indicated a relatively smaller area, with a west–east direction. Thus, the LTA cases (12 km CONUS simulations) compared better to the observation-based products spatially than the base case. The K2C10W case (with WWLLN) tended to produce less precipitation than the K2C10N case (with NLDN) and both observation-based products. These spatial discrepancies for precipitation in OVC between PRISM and the model cases were reflected by the unique statistical behavior, as displayed in Fig. 3 and discussed in Sect. 4.1. As a likely artifact of excessively activated convection within the 108 km grid cells with a spatial scale much larger than most thunderstorm scales, the HK2C10W case indicated areas of heavy precipitation that were also shown in the observation-based products and the 12 km LTA cases (both K2C10W and K2C10N) at approximately the same locations but with much less spatial extent. To resonate with the large discrepancies in the MB values shown in Fig. 11a among the base cases, the precipitation from HK2C10B and HK2C10B cases is similarly displayed in Fig. 12g and h. The case with Trig1 was clearly more comparable to the CONUS cases than the Trig2 case, in that the precipitation from Trig2 was severely underestimated across the entire U.S. These hemispheric simulations amplified the impact of the trigger options on precipitation during warm months among the base cases, resulting in differences in daily total precipitation of up to 40 % in the U.S. (Fig. S6a). These results underscore the need to carefully set cumulus parameters for the KF scheme in WRF simulations.

The mismatch of the spatial scales between domain resolution and thunderstorms in the 108 km simulations is a limitation of the current LTA scheme that could be improved in future development. In addition to using lightning density to trigger convection, another option is to implement the LTA scheme in the Multiscale Kain–Fritsch (MSKF) scheme (Glotfelty et al., 2019; Zheng et al., 2016), a “scale-aware” variant of KF that refines the convective tendencies based on the grid spacing used in the simulation.

### 5.2 Impact on other meteorological variables

**Figure 13.**
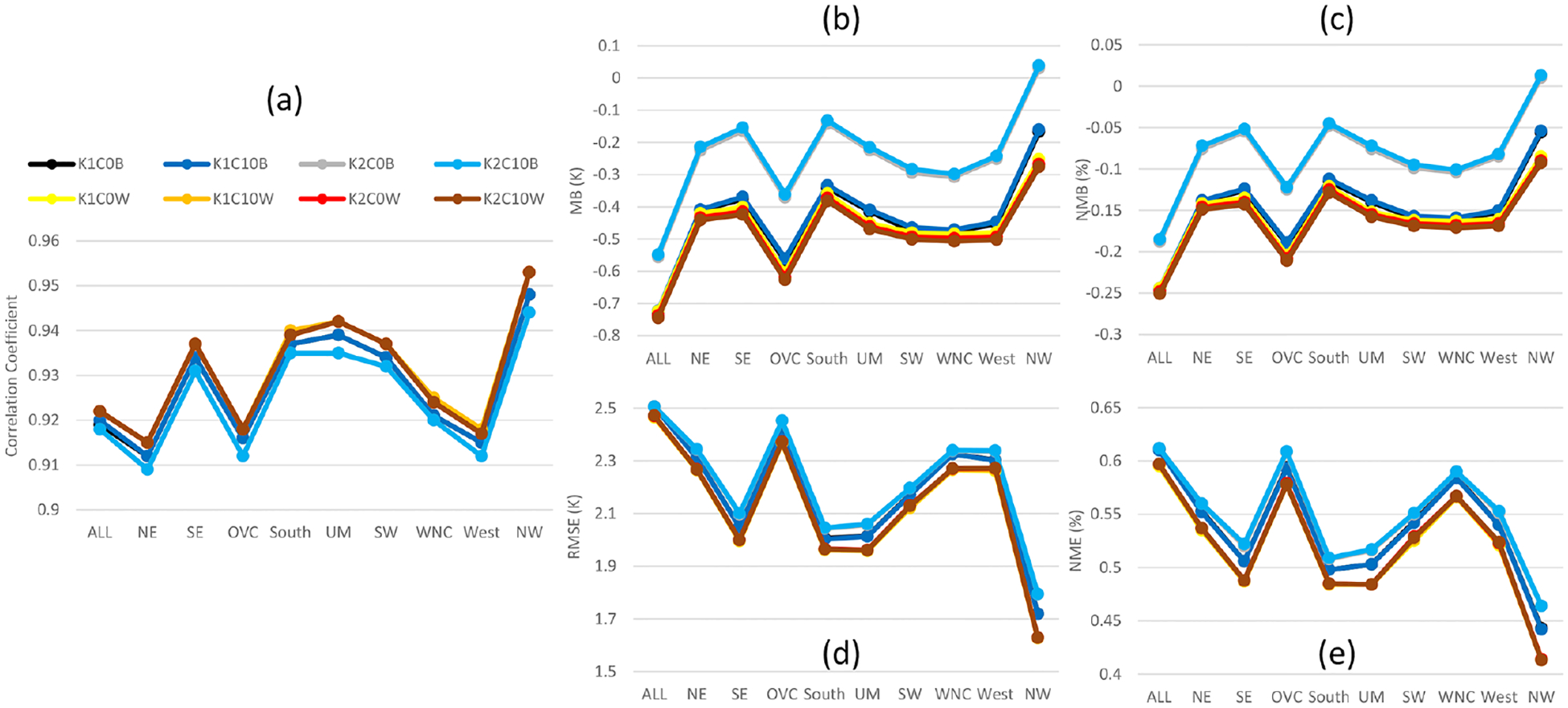
Monthly mean statistics for 2 m temperature from hemispheric base and LTA simulations comparing to surface observations during July 2016. **(a)** Correlation coefficient, **(b)** MB, **(c)** NMB, **(d)** RMSE, and **(e)** NME.

**Figure 14.**
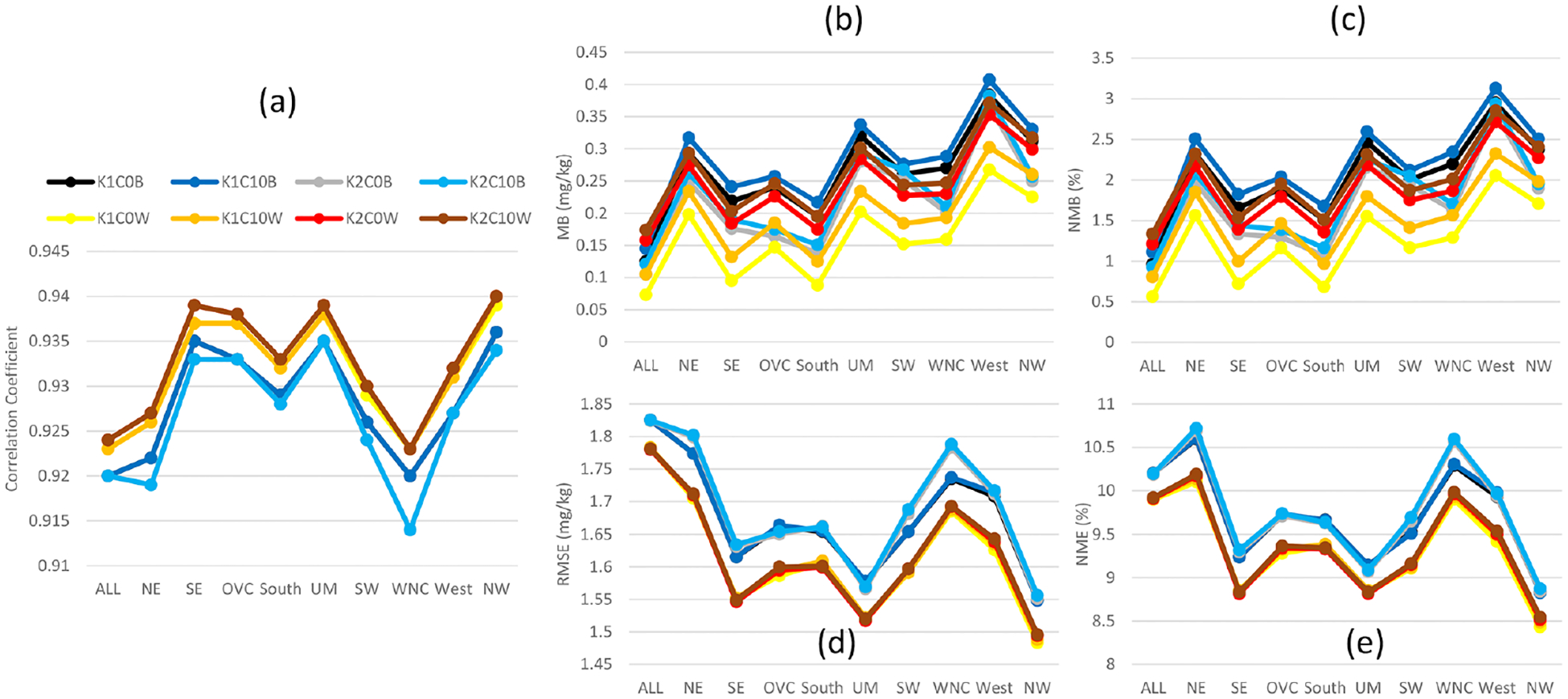
Same as Fig. 13 but for 2 m water vapor mixing ratio.

The impact of the cumulus parameters and LTA scheme on near-surface meteorological variables of the 108 km hemispheric simulations are evaluated like the 12 km CONUS simulations. However, due to the lack of observation data beyond North America, the analysis is mainly focused on the U.S. regions, but all the available data within the hemispheric domain is collectively referred to as “ALL”, regardless of where the data originated. Affected by the coarser domain resolution, all the statistical measures for *T* 2 (Fig. 13) from the hemispheric simulations indicated degradations in model performance relative to the 12 km CONUS domain (Fig. 4). As in the CONUS simulations, the LTA cases increased correlation coefficients and decreased errors (RMSE and NME) compared to the base cases. Like the CONUS simulations, the cumulus parameters minimally affected the LTA cases, while significant deviations were produced among the base cases. Unlike the CONUS simulations where both trigger and cudt contributed to *T* 2 differences, the large differences among the base cases for the hemispheric simulations were attributed to the trigger options. Though all the cases tended to underestimate *T* 2 (contrary to the CONUS simulations where *T* 2 was generally overestimated), among the base cases, greater underestimates were associated with Trig1 than Trig2. The LTA cases uniformly underestimated *T* 2, consistent with the Trig1 base cases. The performance of hemispheric simulations for 2 m water vapor mixing ratio (Fig. 14) resembles *T* 2 in comparison to the CONUS simulations (Fig. 5), which produced smaller correlation coefficients and larger errors and biases (mainly overestimates for both CONUS and hemispheric simulations). Without exception, the LTA cases consistently performed better in terms of correlation coefficients and errors than the base cases. However, different from other meteorological variables, the MB and NMB associated with water vapor mixing ratio are affected by both cumulus parameters (trigger and cudt) for all the model cases (both base cases and LTA cases). The LTA cases with Trig1 performed better than the cases with Trig2, and with the same trigger value, cudt = 0 is preferable to cudt = 10; however, for the base cases, it was the opposite, though with smaller differences. At the 108 km grid spacing, the 10 m wind speed (Fig. S7 in the Supplement) and wind direction (not shown) statistics were comparable among the cumulus parameters and the application of LTA.

## 6 Discussion and recommendations

This study corroborated that the simple observation-based LTA scheme implemented in Heath et al. (2016) improved WRF-simulated precipitation and other near-surface meteorological variables, as evidenced by the simulations over multiple spatial scales and over a longer test period. Testing on a 12 km CONUS domain using lightning flashes from WWLLN instead of NLDN slightly reduced the correlation coefficients and locally increased errors due to the lower detection efficiency of WWLLN. The update of the LTA technique reduced the underestimate of precipitation that was often reported in the application of WRF simulations conducted over the CONUS domain (U.S. EPA, 2019). Changing lightning flash data from NLDN to WWLLN resulted in additional underestimate of precipitation due to fewer lightning flashes in WWLLN than the NLDN dataset. However, when the WWLLN data were used in the hemispheric simulations, the model performance for precipitation over the equatorial regions was significantly improved from significant overestimation in the base cases to slight underestimation in the LTA cases, and the precipitation over land was generally overestimated during the convective season for almost all the selected regions, especially over North America.

The application of LTA in the hemispheric simulations with a 108 km domain exposed a shortcoming of this simple LTA scheme. When the model grid cell is substantially larger than most thunderstorm scales (Murphy and Konrad, 2005), over-triggering of convection within the entire grid cell leads to overestimated precipitation. With the current LTA implementation and the high lightning detection efficiency network, such as NLDN, the 12 km grid spacing is suitable for LTA because thunderstorms often have a radial distance of 1–10 km. When lightning data from low detection efficiency networks (such as WWLLN) are used over finer-resolution domains (≤ 12 km), the NoSuppress option with LTA could balance increasing precipitation while maintaining reasonable levels of uncertainty in the other variables for a more holistic model evaluation. The effect of domain resolution on precipitation simulation with LTA portends further development and improvement in the LTA technique. Two potential developmental directions are to use criteria values of lightning flash density dependent on grid resolution to trigger deep convection and/or to implement the LTA scheme in the MSKF scheme in WRF to adapt to different simulation scales. Preliminary experimentation on the 108 km scale (not shown) suggests that MSKF could improve these comparisons with observations (compared to the KF scheme presented here), including better cloud and precipitation fields (Hogrefe et al., 2021).

The experiment of cumulus parameters (trigger and cudt) associated with the KF scheme was performed for both the CONUS and hemispheric WRF simulations. Results revealed several key behaviors in both the base case simulations and LTA case simulations. First, the base case simulations were sensitive to both trigger and cudt options over the CONUS domain, but only trigger options produced significant variations for the hemispheric simulations. Second, the impact of the cumulus parameters on LTA cases was insignificant for both modeling domains. Separately, the original LTA technique, as described in Heath et al. (2016), showed influence from the cumulus parameters on the LTA cases (Fig. S8 in the Supplement), but after implementing the updated cloud-top height (one model level above −20 °C) and the additional preconditioning shallow convection (see Sect. 2), the fluctuations among the LTA cases were significantly reduced. Third, the most pronounced impact of cumulus parameters was on the amount of precipitation in the base cases. The Trig1 option generated up to a 10 % overestimate of monthly mean daily precipitation over the CONUS, with cudt = 0, and an additional 10 %–15 % overestimate, with cudt = 10, during July 2016. With Trig2, the simulated precipitation became underestimated by about 10 %–15 %, with the cudt contributing to ~ 5 % difference; Cudt10 had fewer underestimates than Cudt0. However, over the hemispheric domain, only the trigger option dramatically affected simulated precipitation; during the summer months (June, July, and August), the Trig2 cases underestimated the mean daily precipitation by up to 40 %, compared to the Trig1 cases that matched the observation-based precipitation products within 10 %. In summary, without LTA, the recommended default values (trigger = 1 and cudt = 0) by WRF documentation remain the best option for both the CONUS and hemispheric simulations to achieve the best model performance, especially for North America, and with LTA, all the options performed equally well.

As one of the most prominent meteorological models, WRF has been widely used in a variety of applications from regional to global scales and from weather and climate studies to air pollution transport in air quality forecast and regulatory compliances. It is important to improve the convective processes (e.g., convective transport of air pollutants matching the times and locations of lightning NO_*x*_ production) to have more accurate precipitation and other meteorological fields with more resources being available, including observational datasets, computing capability, and advanced scheme development. Observation-based data assimilation has been historically proven to be one of the most effective methods to improve model’s performance in time and space. This research is emerging to consider and use the lightning observations that have become available in various formats and scales in the past few decades to improve convection simulations through LTA. Additional networks of lightning observations and more detailed properties associated with the process of lightning discharge are becoming available (such as the strokes per flash, the strength of lightning energy level, and the separation of cloud-to-ground and inter- or intracloud strikes being more accurately quantified, especially with the available satellite lightning products from Geostationary Lightning Mapper (GLM) detection systems borne on the GOES-16 and GOES-17 satellites (Goodman et al., 2013). Accordingly, lightning assimilation techniques will continue to evolve and build upon the research presented here.

## Supplementary Material

Supplement1

## Figures and Tables

**Table 1.** Model cases used in this study. The case names are comprised of elements from the other four columns, which describe the simulation domain (blank is CONUS; H is hemispheric), the version of the Kain–Fritsch trigger that was applied (trigger 1 is K1; trigger 2 is K2), the frequency that the convective properties was updated (every time step is C0; every 10 min is C10), and the lightning data that were assimilated in the simulation (B is base/none, N is NLDN, and W is WWLLN).

Case name	Domain (none or H)	Trigger (K1 or K2)	cudt (C0 or C10)	LTA network (B, N, W)
K1C0B	CONUS	1	0	Base/none
K1C10B	CONUS	1	10	Base/none
K2C0B	CONUS	2	0	Base/none
K2C10B	CONUS	2	10	Base/none
K1C0N	CONUS	1	0	NLDN
K1C10N	CONUS	1	10	NLDN
K2C0N	CONUS	2	0	NLDN
K2C10N	CONUS	2	10	NLDN
K1C0W	CONUS	1	0	WWLLN
K1C10W	CONUS	1	10	WWLLN
K2C0W	CONUS	2	0	WWLLN
K2C10W	CONUS	2	10	WWLLN
HK1C0B	Hemisphere	1	0	Base/none
HK1C10B	Hemisphere	1	10	Base/none
HK2C0B	Hemisphere	2	0	Base/none
HK2C10B	Hemisphere	2	10	Base/none
HK1C0W	Hemisphere	1	0	WWLLN
HK1C10W	Hemisphere	1	10	WWLLN
HK2C0W	Hemisphere	2	0	WWLLN
HK2C10W	Hemisphere	2	10	WWLLN

## Data Availability

The WRF model is available for download through the WRF GitHub (https://github.com/wrf-model/WRF/tree/v4.1.1, last access: 10 November 2022) (University Corporation for Atmospheric Research, 2022a). The LTA code is not publicly available yet, but interested users can contact the corresponding author to acquire the source code. The raw lightning flash observation data can be purchased through Vaisala Inc. (https://www.vaisala.com/en/products/systems/lightning-detection, last access: 7 November 2022) (Vaisala Inc., 2022), and the WWLLN raw data are also available for purchase at http://wwlln.net (last access: 7 November 2022) (University of Washington, 2022). The immediate data, except the lightning flash data behind the figures, are available from https://doi.org/10.5281/zenodo.6493145 (Kang et al., 2022). PRISM precipitation data for the United States are retrieved from https://climatedataguide.ucar.edu/climate-data/ (last access: 7 November 2022) (University Corporation for Atmospheric Research, 2022b), and the CPC Global Unified Precipitation data provided by the NOAA/OAR/ESRL PSL, Boulder, Colorado, USA, from https://psl.noaa.gov/data/gridded/data.cpc.globalprecip.html (last access: 7 November 2022) (National Oceanic and Atmospheric Administration, 2022). The IMERG data were provided by the NASA/Goddard Space Flight Center’s Precipitation Measurement Missions (PMM) Science Team and Precipitation Processing System (PPS), which develop and compute the IMERG as a contribution to GPM, and are archived at the NASA Goddard Earth Sciences Data and Information Services Center (GES DISC).
