# Supplementary material for: Lightning assimilation in the WRF model (Version 4.1.1): technique updates and assessment of the applications from regional to hemispheric scales
Source: Geosci Model Dev. Author manuscript; Available in PMC 2025 Jan 27. (PMC11770413; doi:10.5194/gmd-15-8561-2022)
Supplement: Supplement1 [file NIHMS2037137-supplement-Supplement1.pdf]

Supplement of Geosci. Model Dev., 15, 8561–8579, 2022
https://doi.org/10.5194/gmd-15-8561-2022-supplement
© Author(s) 2022. CC BY 4.0 License.

Geoscientific Model Development
Open Access

Supplement of

Lightning assimilation in the WRF model (Version 4.1.1): technique updates and assessment of the applications from regional to hemispheric scales

Daiwen Kang et al.

Correspondence to: Daiwen Kang (kang.daiwen@epa.gov)

The copyright of individual parts of the supplement might differ from the article licence.

Table S1. An example of namelist input configuration file for CONUS WRF simulations

```
&time_control
 start_year                          = 2016,
 start_month                         = 3,
 start_day                           = 20,
 start_hour                          = 00,
 start_minute                        = 00,
 start_second                        = 00,
 end_year                            = 2016,
 end_month                           = 08,
 end_day                             = 01,
 end_hour                            = 00,
 end_minute                          = 00,
 end_second                          = 00,
 interval_seconds                    = 10800,
 input_from_file                     = .true.,
 fine_input_stream                   = 0,
 history_interval                    = 60,
 frames_per_outfile                  = 24,
 restart                             = .false.,
 cycling                             = .false.,
 restart_interval                    = 14400,
 reset_simulation_start              = .false.,
 write_hist_at_0h_rst                = .true.,
 override_restart_timers             = .true.
 io_form_history                     = 2
 io_form_restart                     = 2
 io_form_input                       = 2
 io_form_boundary                    = 2
 debug_level                         = 0
 diag_print                          = 2,
 io_form_auxinput2                   = 2,
 io_form_auxinput4                   = 2,
 io_form_auxinput8                   = 2,
 auxinput8_inname                    = 'NLDN_WRF12km_30min_2016Sum.ncf'
 frames_per_auxinput8                = 7344,
 auxinput8_interval_m                = 30
 auxinput8_end_h                     = 9999,
 auxinput1_inname                    = "met_em.d<domain>.<date>"
 auxinput4_inname                    = "wrflowinp_d<domain>"
 auxinput4_interval                  = 180,
 auxinput4_end_h                     = 9999999
 force_use_old_data                  =.true.
 iofields_filename                   = "output.var.txt"
 /

 &domains
 time_step                           = 60,
 time_step_fract_num                 = 0,
 time_step_fract_den                 = 1,
 max_dom                             = 1,
 s_we                                = 1,
 e_we                                = 472,
 s_sn                                = 1,
 e_sn                                = 312,
 s_vert                              = 1,
 e_vert                              = 36,
 p_top_requested                     = 5000,
 eta_levels                          = 1.000, 0.9975, 0.995, 0.990, 0.985,
                                       0.980, 0.970, 0.960, 0.950,
                                       0.940, 0.930, 0.920, 0.910,
                                       0.900, 0.880, 0.860, 0.840,
                                       0.820, 0.800, 0.770, 0.740,
                                       0.700, 0.650, 0.600, 0.550,
                                       0.500, 0.450, 0.400, 0.350,
                                       0.300, 0.250, 0.200, 0.150,
                                       0.100, 0.050, 0.000
```

```
num_metgrid_levels                  = 40,
num_metgrid_soil_levels             = 4,
dx                                  = 12000,
dy                                  = 12000,
force_sfc_in_vinterp                = 1,
interp_type                         = 2,
extrap_type                         = 2,
t_extrap_type                       = 2,
use_levels_below_ground             = .true.,
use_surface                         = .true.,
lagrange_order                      = 2,
zap_close_levels                    = 500.,
lowest_lev_from_sfc                 = .false.,
sfcp_to_sfcp                        = .false.,
smooth_cg_topo                      = .false.,
use_tavg_for_tsk                    = .false.,
rh2qv_wrt_liquid                    = .true.,
grid_id                             = 1,
parent_id                           = 0,
i_parent_start                      = 0,
j_parent_start                      = 0,
parent_grid_ratio                   = 1,
parent_time_step_ratio              = 1,
feedback                            = 0,
smooth_option                       = 0,
use_adaptive_time_step              = .false.,
step_to_output_time                 = .true.,
target_cfl                          = 1.2,
max_step_increase_pct               = 5,
starting_time_step                  = 60,
max_time_step                       = -1,
min_time_step                       = -1,
tile_sz_x                           = 0,
tile_sz_y                           = 0,
numtiles                            = 1,
nproc_x                             = -1,
nproc_y                             = -1,
/

&physics
mp_physics                          = 10,
mp_zero_out                         = 2,
mp_zero_out_thresh                  = 1.0e-8,
ra_lw_physics                       = 4,
ra_sw_physics                       = 4,
radt                                = 20,
co2tf                               = 1,
sf_sfclay_physics                   = 7,
num_soil_layers                     = 2,
pxlsm_smois_init                    = 0,
pxlsm_modis_veg                     = 1,
sf_surface_physics                  = 7,
sf_urban_physics                    = 0,
bl_pbl_physics                      = 7,
bldt                                = 0,
cu_physics                          = 1,
cudt                                = 0,
kfeta_trigger                       = 1,
ltg_assim                           = .true.,
suppress_opt                        = 2,
prec_acc_dt                         = 60,
isfflx                              = 1,
ifsnow                              = 1,
icloud                              = 1,
cu_rad_feedback                     = .true.,
surface_input_source                = 1,
num_land_cat                        = 40,
num_soil_cat                        = 16,
sst_update                          = 1,
```

```
maxiens                             = 1,
maxens                              = 3,
maxens2                             = 3,
maxens3                             = 16,
ensdim                              = 144,
seaice_threshold                    = 100,
usemonalb                           = .false.,
rdmaxalb                            = .true.,
rdlai2d                             = .false.,
sst_skin                            = 0,
tmn_update                          = 0,
lagday                              = 1,
fractional_seaice                   = 0,
iz0tlnd                             = 0,
bucket_mm                           = -1,
bucket_J                            = -1,
slope_rad                           = 1,
topo_shading                        = 1,
shadlen                             = 25000.,
do_radar_ref                        = 1,
grav_settling                       = 0,
/

&fdda
grid_fdda                           = 1,
grid_sfdda                          = 1,
pxlsm_soil_nudge                    = 1,
sgfdda_inname                       = "wrfsfdda_d<domain>",
sgfdda_end_h                        = 999999,
sgfdda_interval_m                   = 180,
gfdda_inname                        = "wrffdda_d<domain>",
gfdda_end_h                         = 999999,
gfdda_interval_m                    = 180,
fgdt                                = 0,
if_no_pbl_nudging_uv                = 1,
if_no_pbl_nudging_t                 = 1,
if_no_pbl_nudging_q                 = 1,
if_zfac_uv                          = 0,
 k_zfac_uv                          = 13,
if_zfac_t                           = 0,
 k_zfac_t                           = 13,
if_zfac_q                           = 0,
 k_zfac_q                           = 13,
guv                                 = 0.0001,
gt                                  = 0.0001,
gq                                  = 0.00001,
guv_sfc                             = 0.0000,
gt_sfc                              = 0.0000,
gq_sfc                              = 0.0000,
if_ramping                          = 1,
dtramp_min                          = 60.0,
io_form_gfdda                       = 2,
rinblw                              = 250.0
/

&dynamics
hybrid_opt                          = 2,
rk_ord                              = 3,
w_damping                           = 1,
diff_opt                            = 1,
km_opt                              = 4,
diff_6th_opt                        = 2,
diff_6th_factor                     = 0.12,
damp_opt                            = 3,
base_temp                           = 290.
zdamp                               = 5000.,
dampcoef                            = 0.20,
khdif                               = 0,
kvdif                               = 0,
```

```
 smdiv                                = 0.1,
 emdiv                                = 0.01,
 epssm                                = 0.1,
 non_hydrostatic                      = .true.,
 top_lid                              = .false.,
 mix_full_fields                      = .false.,
 mix_isotropic                        = 0,
 mix_upper_bound                      = 0.1,
 tke_drag_coefficient                 = 0.0,
 tke_heat_flux                        = 0.0,
 h_mom_adv_order                      = 5,
 v_mom_adv_order                      = 3,
 h_sca_adv_order                      = 5,
 v_sca_adv_order                      = 3,
 moist_adv_opt                        = 2,
 scalar_adv_opt                       = 2,
 chem_adv_opt                         = 1,
 tke_adv_opt                          = 2,
 time_step_sound                      = 0,
 fft_filter_lat                       = 91,
 gwd_opt                              = 0,
 use_theta_m                          = 0,
 /

 &bdy_control
 spec_bdy_width                       = 5,
 spec_zone                            = 1,
 relax_zone                           = 4,
 specified                            = .true.,
 spec_exp                             = 0.0,
 nested                               = .false.,
 /

 &grib2
 /

 &namelist_quilt
 nio_tasks_per_group = 0,
 nio_groups = 1,
/
```

Table S2. An example of namelist input configuration file for hemispheric WRF simulations

```
&time_control
 start_year                          = 2016
 start_month                         = 01
 start_day                           = 31
 start_hour                          = 00
 start_minute                        = 00,
 start_second                        = 00,
 end_year                            = 2017
 end_month                           = 01
 end_day                             = 01
 end_hour                            = 00
 end_minute                          = 00,
 end_second                          = 00,
 interval_seconds                    = 21600
 input_from_file                     = .true.,
 history_interval                    = 60,
 frames_per_outfile                  = 24,
 restart                             = true,
 restart_interval                    = 1440,
 reset_simulation_start              = .false.,
 write_hist_at_0h_rst                = .true.,
 override_restart_timers             = .true.
 io_form_history                     = 2
 io_form_restart                     = 2
 io_form_input                       = 2
 io_form_boundary                    = 2
 io_form_auxinput2                   = 2
 io_form_auxinput4                   = 2
 io_form_auxinput8                   = 2,
 auxinput8_inname                    = 'WWLLN_Hemi_30min_20161231.ncf'
 frames_per_auxinput8                = 48,
 auxinput8_interval_m                = 30
 auxinput8_end_h                     = 9999,
 auxinput4_inname                    = "wrflowinp_d01"
 auxinput4_interval                  = 360
 auxinput4_end_h                     = 999999999
 auxinput1_inname                    = "met_em.d01.<date>"
 iofields_filename                   = "output.var.txt"
 /

 &domains
 time_step                           = 180,
 time_step_fract_num                 = 0,
 time_step_fract_den                 = 1,
 max_dom                             = 1,
 s_we                                = 1,
 e_we                                = 200,
 s_sn                                = 1,
 e_sn                                = 200,
 s_vert                              = 1,
 e_vert                              = 45,
 p_top_requested                     = 5000,
 eta_levels                          = 1.000, 0.9975, 0.9946, 0.9913, 0.9875,
                                       0.9831, 0.9781, 0.9723, 0.9657, 0.958,
                                       0.9492, 0.9391, 0.9275, 0.9141, 0.8987,
                                       0.881, 0.8607, 0.8373, 0.8104, 0.7795,
                                       0.7439, 0.7066, 0.6693, 0.632, 0.5946,
                                       0.5573, 0.52, 0.4827, 0.4454, 0.4081,
                                       0.3708, 0.3352, 0.3013, 0.269, 0.2383,
                                       0.2089, 0.181, 0.1543, 0.1289, 0.1047,
                                       0.0816, 0.0596, 0.0386, 0.0186, 0.000
 num_metgrid_levels                  = 40,
 dx                                  = 108000,
 dy                                  = 108000,
```

```
grid_id                              = 1,
parent_id                            = 0,
i_parent_start                       = 0,
j_parent_start                       = 0,
parent_grid_ratio                    = 1,
parent_time_step_ratio               = 1,
feedback                             = 1,
smooth_option                        = 0,
/

&physics
mp_physics                           = 10,
mp_zero_out                          = 2,
ra_lw_physics                        = 4,
ra_sw_physics                        = 4,
radt                                 = 20,
sf_sfclay_physics                    = 7,
sf_surface_physics                   = 7,
bl_pbl_physics                       = 7,
bldt                                 = 0,
cu_physics                           = 1,
kfeta_trigger                        = 2,
ltg_assim                            = .true.,
suppress_opt                         = 2,
cu_rad_feedback                      = .true.,
cudt                                 = 0,
prec_acc_dt                          = 60,
isfflx                               = 1,
ifsnow                               = 1,
icloud                               = 1,
surface_input_source                 = 1,
num_soil_layers                      = 2,
sst_update                           = 1,
pxlsm_smois_init                     = 0,
slope_rad                            = 1,
topo_shading                         = 0,
shadlen                              = 25000.,
num_land_cat                         = 20,
/

&fdda
grid_fdda                            = 1,
grid_sfdda                           = 1,
pxlsm_soil_nudge                     = 1,
sgfdda_inname                        = "wrfsfdda_d01",
sgfdda_end_h                         = 9999999,
sgfdda_interval_m                    = 360,
gfdda_inname                         = "wrffdda_d<domain>",
gfdda_end_h                          = 9999999,
gfdda_interval_m                     = 360,
fgdt                                 = 0,
if_no_pbl_nudging_uv                 = 1,
if_no_pbl_nudging_t                  = 1,
if_no_pbl_nudging_q                  = 1,
if_zfac_uv                           = 0,
 k_zfac_uv                           = 13,
if_zfac_t                            = 0,
 k_zfac_t                            = 13,
if_zfac_q                            = 0,
 k_zfac_q                            = 13,
guv                                  = 0.0001,
gt                                   = 0.0001,
gq                                   = 0.00001,
guv_sfc                              = 0.0000,
gt_sfc                               = 0.0000,
gq_sfc                               = 0.0000,
if_ramping                           = 1,
dtramp_min                           = 60.0,
io_form_gfdda                        = 2,
```

```
rinblw                              = 250.0
/

&dynamics
hybrid_opt                          = 2,
w_damping                           = 1,
diff_opt                            = 1,
km_opt                              = 4,
kvdif                               = 0,
non_hydrostatic                     = .true.,
moist_adv_opt                       = 2,
tke_adv_opt                         = 2,
scalar_adv_opt                      = 2,
use_theta_m                         = 1,
/

&bdy_control
spec_bdy_width                      = 5,
spec_zone                           = 1,
relax_zone                          = 4,
specified                           = .true.,
nested                              = .false.,
/

&grib2
/

&namelist_quilt
nio_tasks_per_group = 0,
nio_groups = 1,
/
diff_6th_opt                        = 2,
diff_6th_factor                     = 0.12,
damp_opt                            = 3,
base_temp                           = 290.
zdamp                               = 5000.,
dampcoef                            = 0.05,
khdif                               = 0,
```

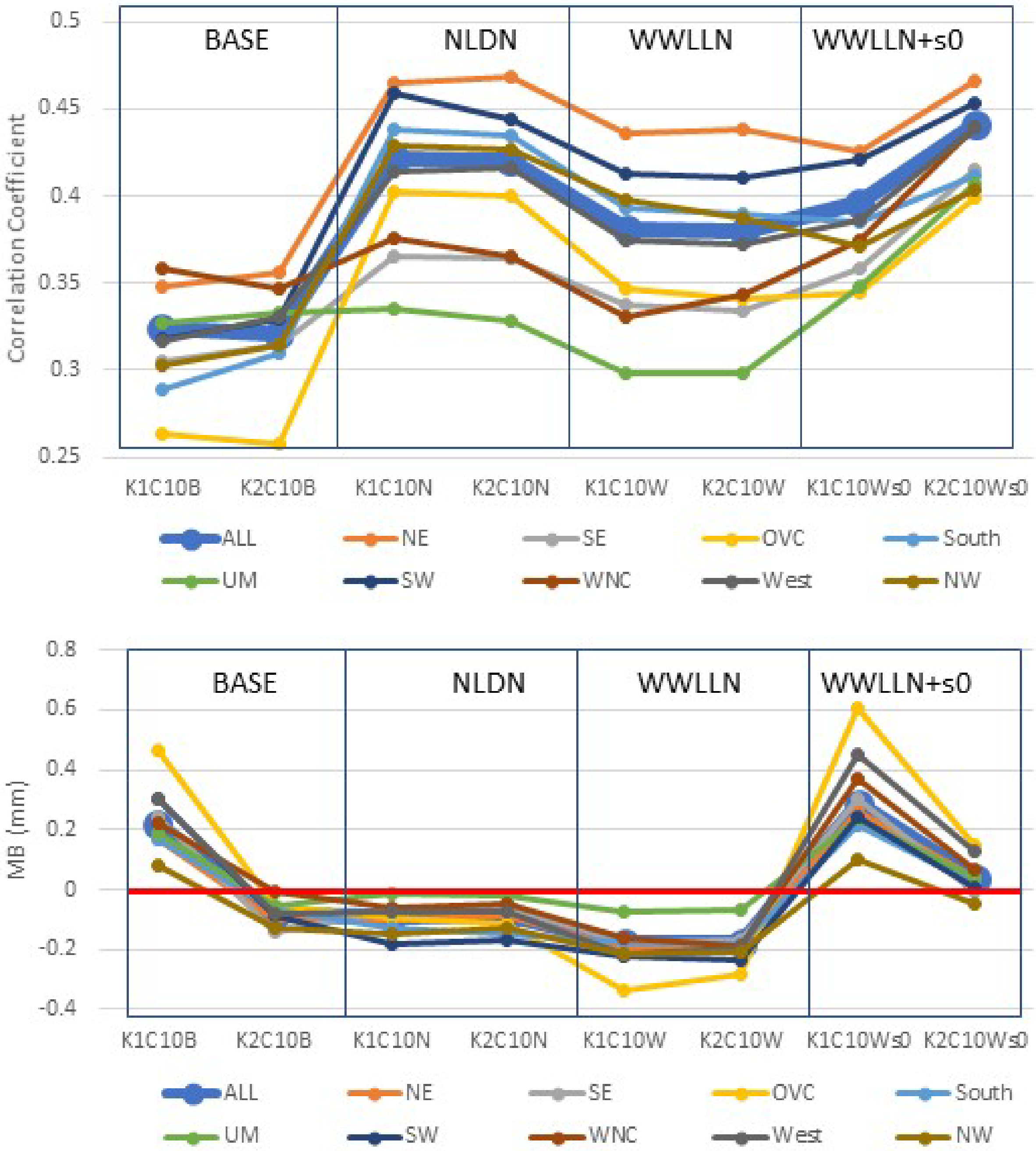

Figure S1. Monthly mean statistics (correlation coefficients and MB) for precipitation from BASE and LTA simulations for selected cases comparing to the values from PRISM for the modeling domain and the climatological regions over the CONUS, respectively, during July 2016. In each plot, there are four sets of simulations (BASE, LTA with NLDN, LTA with WWLLN, and LTA with WWLLN+s0) and each having two cases from the combinations of cumulus parameters (trigger values of 1 and 2 with cult = 10). The BASE, NLDN, and WWLLN cases are the same as in Figure 3, but the WWLLN+s0 case differs from WWLLN in that the "Suppress" option in WWLLN is "ShallowOnly" but in WWLLN+so it is "NoSuppress", i.e., when lightning is not present, KF runs as usual (the same as in BASE cases).

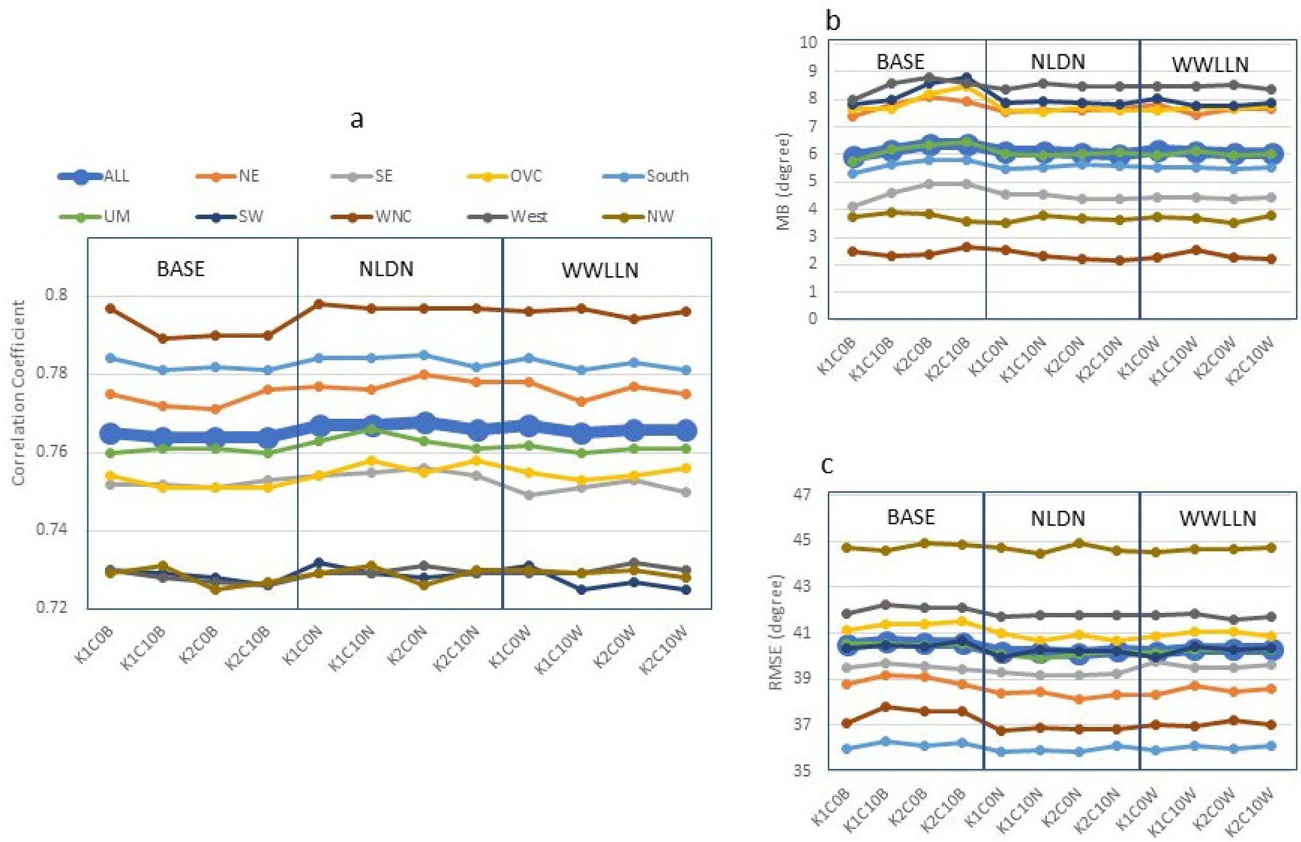

Figure S2. Same as Figure 4, but for 10-m wind direction with correlation coefficient, MB, and RMSE.

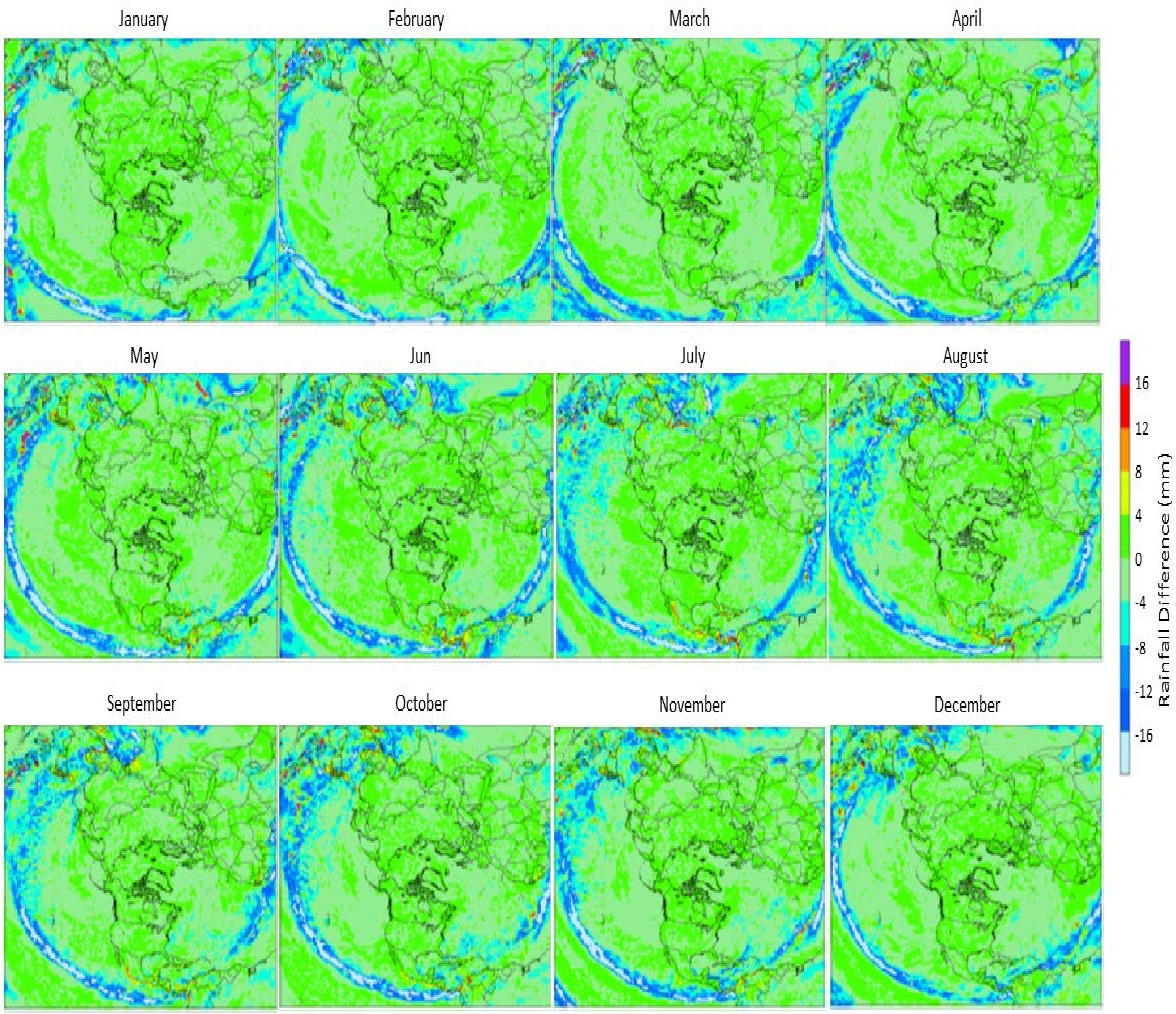

Figure S3. Monthly rainfall difference between simulations with LTA (HK1C0W) and without LTA (HK1C0B) over the northern hemisphere (HK1C0W – HK1C0B).

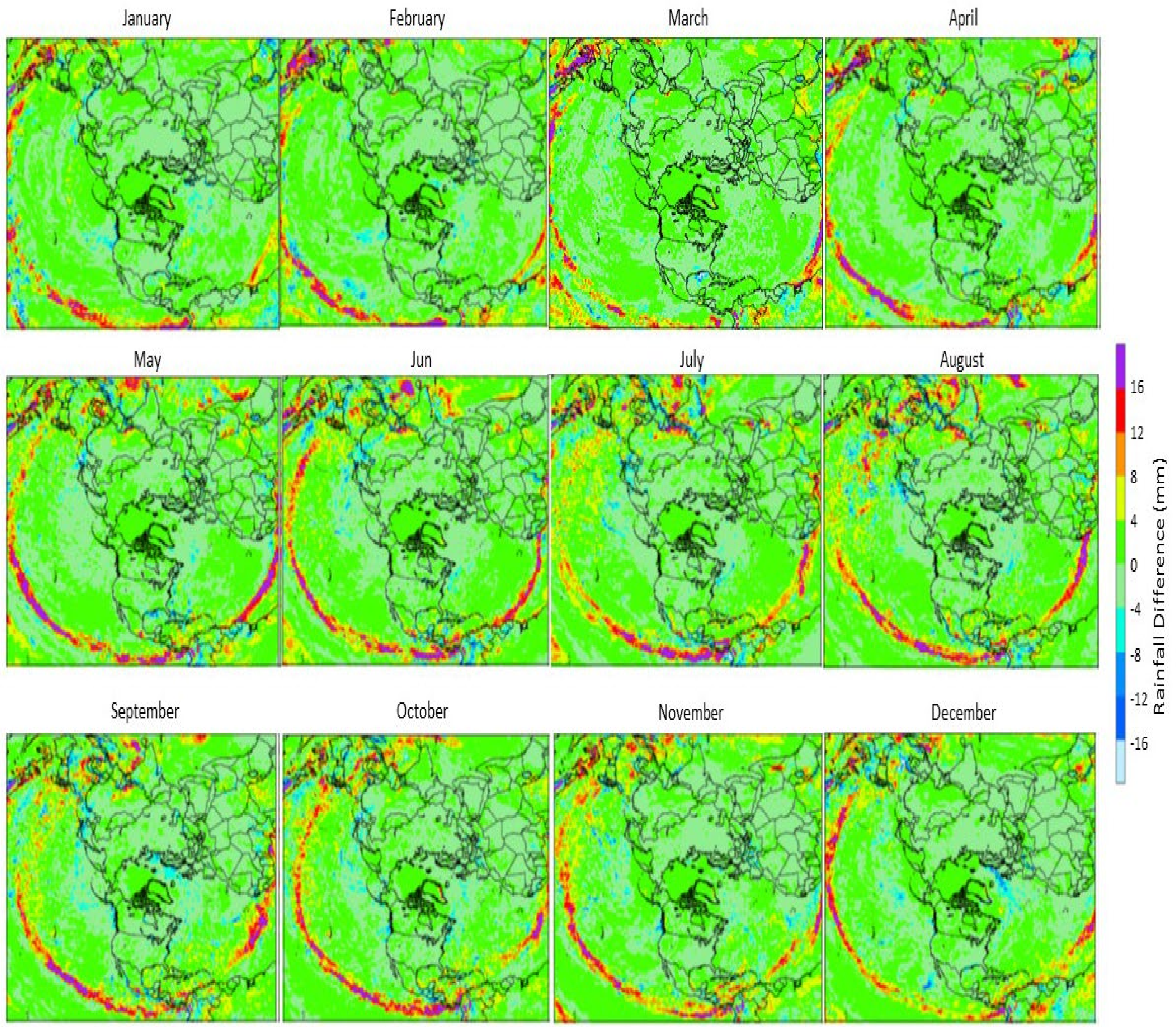

Figure S4. Monthly rainfall difference between the Base Case (HK1C0B) and The IMERG rainfall over the northern hemisphere (HK1C0B – IMERG).

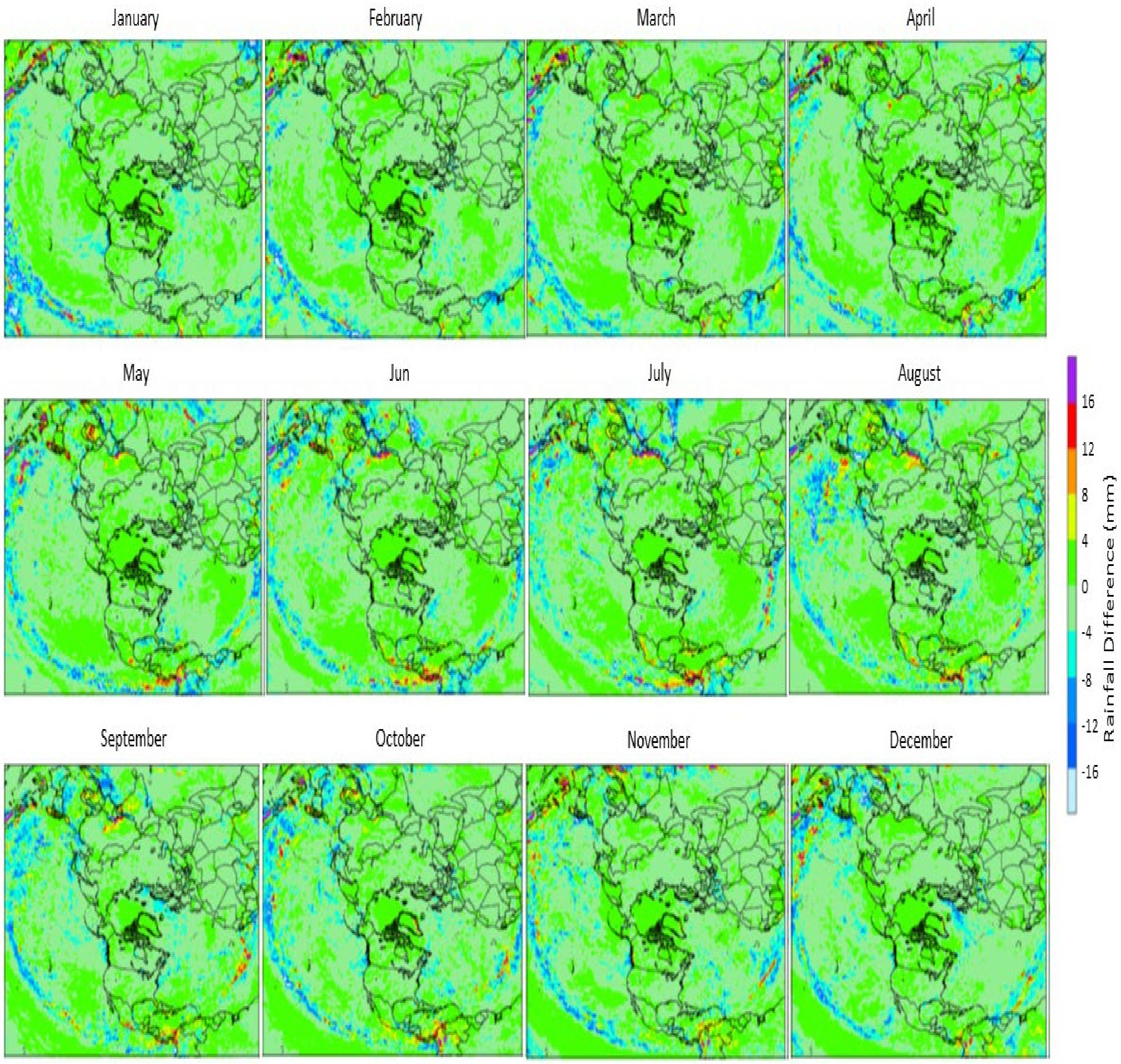

Figure S5. Mean daily rainfall difference by month between the LTA Case (HK1C0W) and The IMERG rainfall over the northern hemisphere (HK1C0W – IMERG).

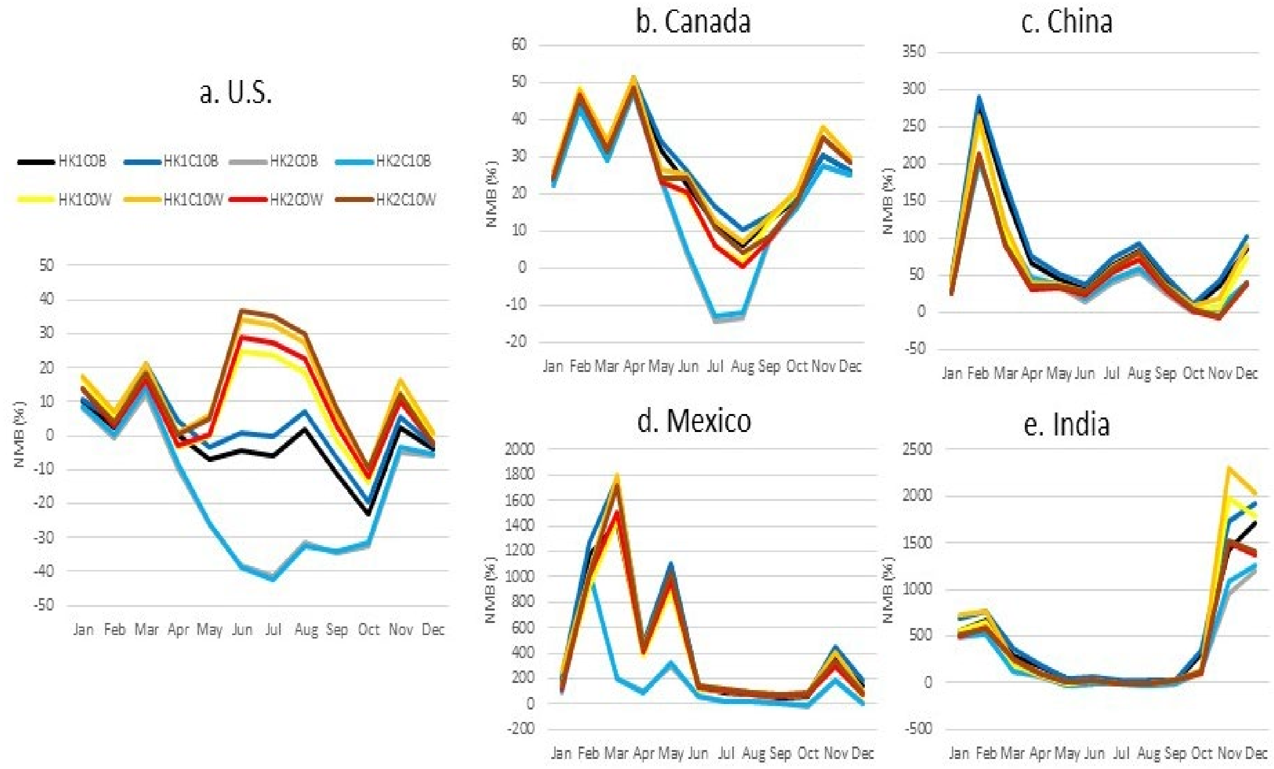

Figure S6. Same as Figure 8, but for NMB.

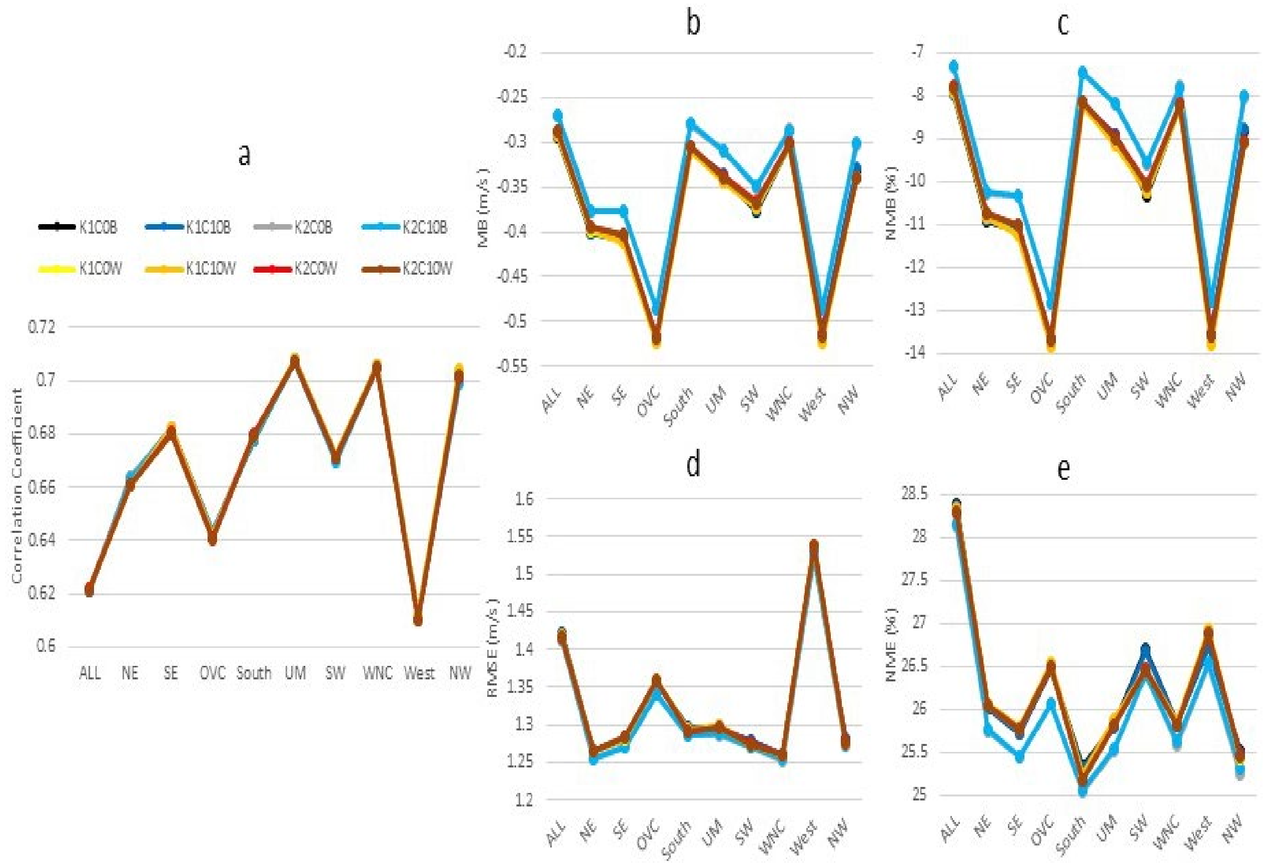

Figure S7. Same as Figure 12, but for 10-m wind speed.

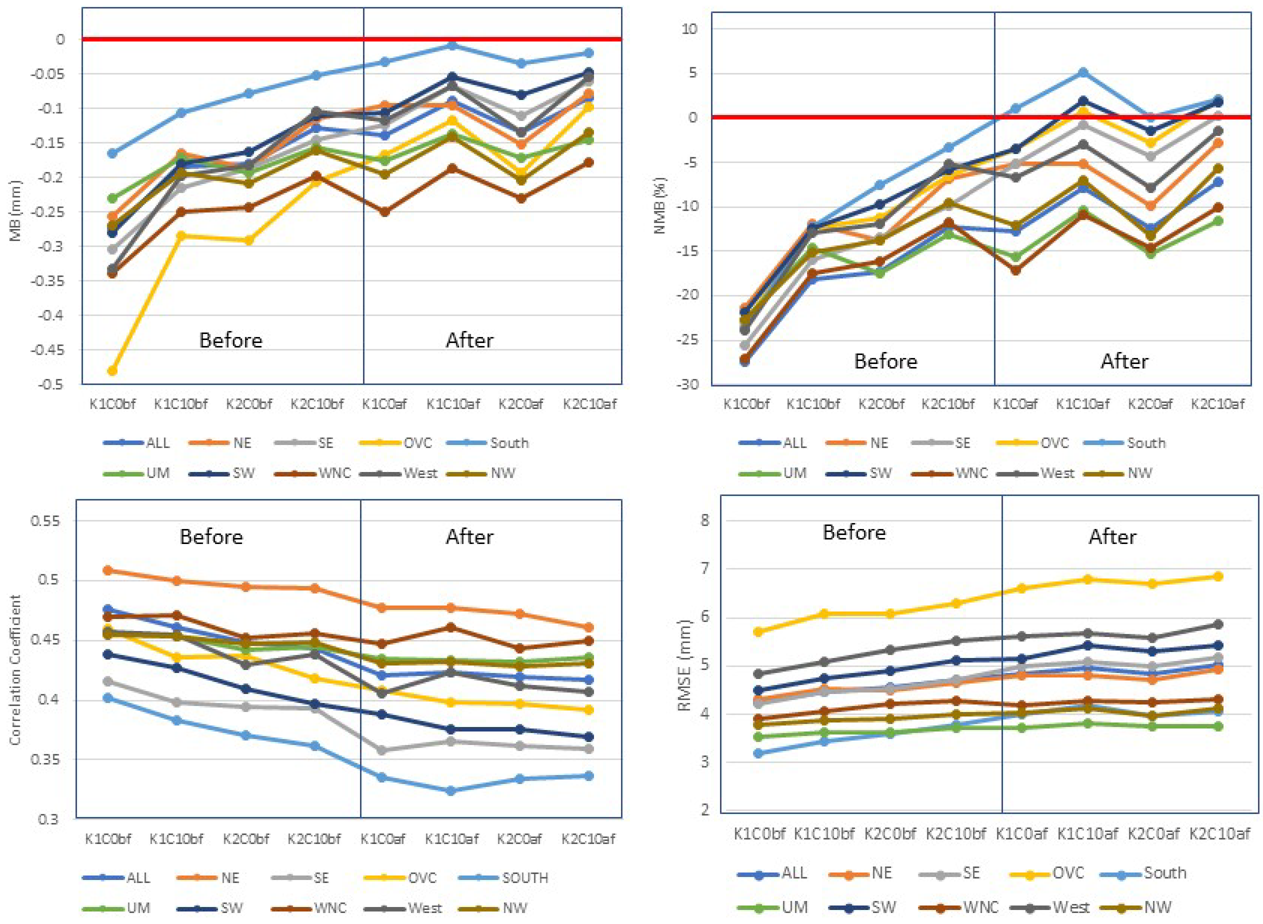

Figure S8. The change of precipitation statistics before and after the LTA updates. The bf stands for before and af stands for after with the case names, and the other part of the names are the same as earlier figures.
